# A Hollow Shell‐Lattice Soft Robot in Flexible Pipelines with Flowing Fluids

**DOI:** 10.1002/advs.202414882

**Published:** 2025-03-27

**Authors:** Di Guo, Yiqiang Wang, Zhan Kang

**Affiliations:** ^1^ State Key Laboratory of Structural Analysis Optimization and CAE Software for Industrial Equipment Dalian University of Technology Dalian 116024 China

**Keywords:** flowing fluids, hollow body, lattice shells, pipeline inspection, soft robots

## Abstract

Pipeline‐crawling soft robots are increasingly preferable for effective inspection and maintenance of flexible pipes. However, most existing robots occupy the pipe cross‐sections, disrupting the normal operation of the working systems. In this study, an innovative shell‐lattice soft robot is designed for crawling in pipes with fluid flows. The robot features a hollow body with a pneumatic actuator in the middle and two lattice shells at the head and tail parts. It enables earthworm‐like locomotion through the implementation of opposite radial deformations in the two lattice shells. The hollow body architecture ensures unimpeded fluid flow during its crawling, even when mixed with solid impurities. Moreover, the surface‐to‐surface contact of the robot with the pipeline walls confers superior load‐carrying capability, facilitating the transport of devices necessary for inspection and maintenance tasks. The robot is also capable of traversing various pipes with different frictional coefficients, irregular cross‐sectional shapes, and varying curvatures, and can support untethered operation. Finally, potential applications of this robot in obstructed concealed pipes and disturbed offshore pipes are demonstrated. By leveraging advanced fabrication techniques, smart materials, and propulsion methods, it is anticipated that the designed robot may show significant scalability and applicability across diverse domains, including healthcare, aviation, and gas‐and‐oil transportation.

## Graphical abstract

This study proposes a novel shell‐lattice robot with hollow bodies, capable of crawling through pipes with flowing fluids of high velocities and viscosities and mixed with impurities. The robot also features capabilities of crawling with high payloads, in curved pipes, and unthethered crawling. These unique properties make this robot high promising for the inspection and maintenance of concealed and offshore pipelines.

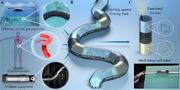

## 1 Introduction

**Figure 1:**
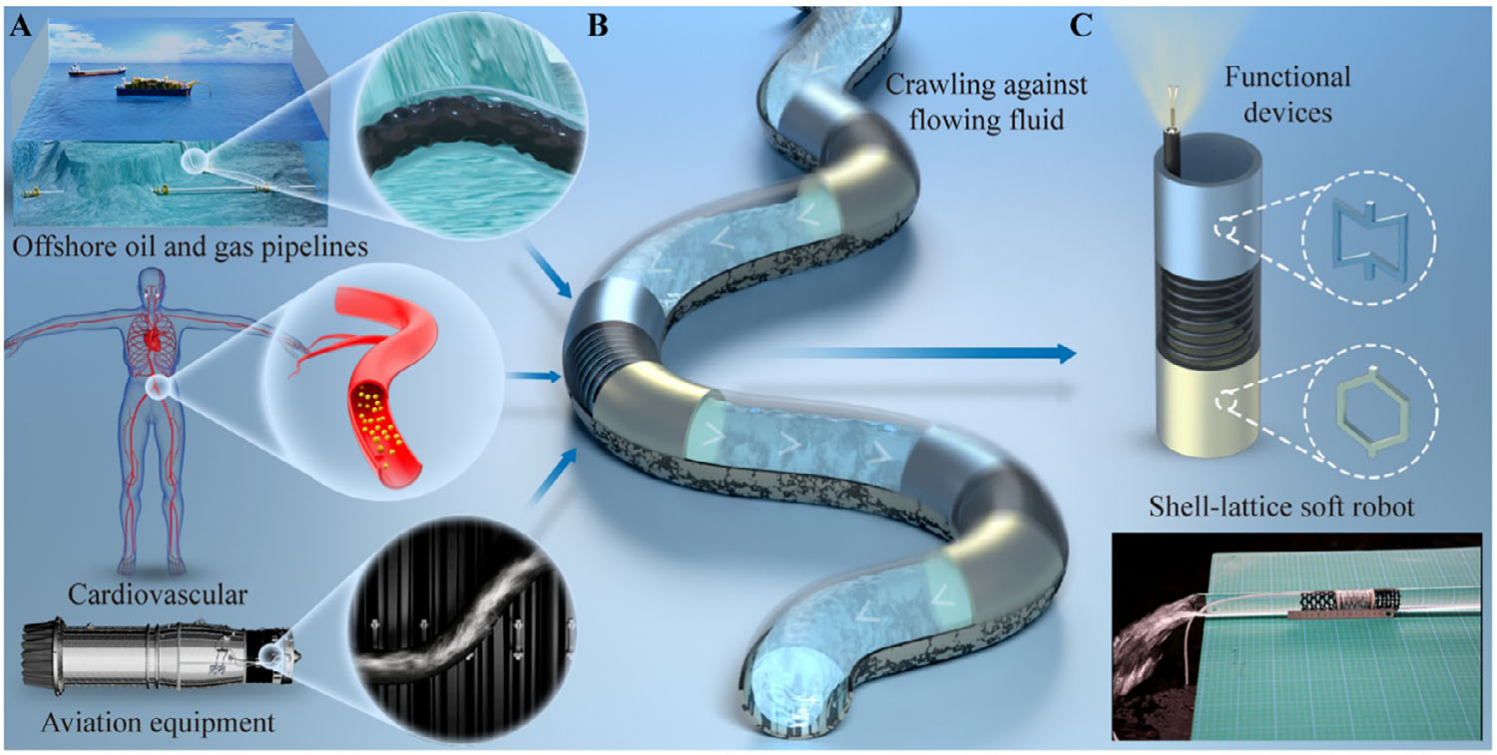
Schematic illustration of the necessity to design shell‐lattice soft robots for inspection and maintenance of the pipelines with flowing fluids. A) The importance of pipelines in three scenarios. B) A crawling process of hollow robots in pipelines with fluids. C) Our proposed shell‐lattice soft robot with functional devices.

Pipelines are extensively used in diverse applications, including oil and gas transportation systems and turbine engines (**Figure** **1**A).^[^ ^1^, ^2^ ^]^ Particularly, flexible pipelines are indispensable components of the human body, including those in the cardiovascular and respiratory systems (Figure 1A).^[^ ^3^, ^4^, ^5^ ^]^ They play critical roles in transporting substances, chemicals, or energy. However, they may suffer from degradation or damage during their service life, including wall corrosion, surface contamination, rupture, and blockage.^[^ ^6^, ^7^, ^8^ ^]^ These issues may lead to serious consequences such as environmental pollution, economic losses, and even human casualties. Therefore, it is crucial to conduct pipeline inspection and maintenance to ensure pipeline integrity and functionality.

Pipeline‐crawling robots have gained popularity for pipeline inspection and maintenance due to their ability to move in confined spaces.^[^ ^8^, ^9^, ^10^, ^11^, ^12^, ^13^ ^]^ Despite well‐developed rigid robots,^[^ ^14^, ^15^, ^16^ ^]^ soft robots are increasingly favored for use in flexible pipelines.^[^ ^17^, ^18^ ^]^ Their comparable stiffness to that of the flexible pipes effectively prevents puncture damage,^[^ ^19^, ^20^ ^]^ and their soft bodies are better suited to accommodate pipe deformations under various conditions.^[^ ^21^, ^22^, ^23^, ^24^, ^25^, ^26^, ^27^ ^]^ To date, numerous types of pipeline‐crawling soft robots have been proposed. For instance, pneumatic soft robots featuring multiple air chambers^[^ ^28^, ^29^, ^30^, ^31^, ^32^, ^33^ ^]^ allow for independent actuation of each chamber, enabling them to crawl through pipes in an earthworm‐like locomotion mode. By varying the actuation sequence and pressure magnitudes, specified deformation modes can be achieved,^[^ ^34^, ^35^ ^]^ making these robots suitable for navigating curved and multi‐branch pipes. In comparison, robots with a single pneumatic actuator facilitate the actuation and control systems, but rely on passive deformation of their body parts for crawling.^[^ ^36^, ^37^ ^]^ Besides pneumatic actuation, other excitation methods have also been explored. For instance, a dielectric‐elastomer‐actuated soft robot has been designed for operation in aircraft engine pipelines with sub‐centimeter dimensions.^[^ ^1^ ^]^ Moreover, strip‐like bodies can roam in multimodal locomotion when remote magnetic fields are applied,^[^ ^3^, ^23^ ^]^ while hexagonal lattice bodies realize crawling in a rotational mode.^[^ ^38^ ^]^

Two fundamental functionalities must be realized when designing robots for crawling in pipes with flowing fluids (Figure 1B). First, the inspection process needs to ensure the normal operation conditions of the pipelines, as any interruption in flowing fluids can lead to significant complications. For instance, in the cardiovascular system, disrupted blood flow can pose serious risks to life. However, many existing robots occupy the entire cross‐section of the pipes,^[^ ^30^ ^]^ thereby impeding the smooth fluid flow and substance transportation. Additionally, the high resistance from flowing fluids further complicates the crawling of these robots. Second, the robots need to be equipped with devices for effective pipeline inspections and maintenance, which requires both sufficient stiffness and adequate internal space. While soft strips provide some space,^[^ ^23^ ^]^ they often lack the necessary stiffness to support the required devices. Furthermore, their rotational motion may hinder precise position control,^[^ ^22^, ^23^, ^38^ ^]^ which is crucial for identifying pipeline issues and cleaning obstacles at specific positions. Therefore, achieving these functionalities necessitates new design concepts of the robot body.

In this work, we propose a shell‐lattice soft robot that can crawl through pipelines with flowing fluids. Its main body consists of three hollow cylinders, in which the central part serves as a pneumatic actuator while the head and tail parts engage the inner walls of the pipe to provide sufficient friction for crawling (Figure 1C). Here, lattice shells are employed in the head and tail sections to facilitate opposite radial deformations under the same actuation forces. This hollow lattice design endows our robot with several distinctive advantages compared to other existing robot designs. First, the large hollow cross‐sectional area guarantees a smooth flow of fluids within the pipelines during the robot crawling, even in the presence of solid impurities. Second, thin‐walled structures effectively minimize resistance to flowing fluids as the robot moves forward (Note S1 and Figure S1A,B, Supporting Information). Third, the hollow design can accommodate essential devices for pipeline inspection and maintenance, such as cameras and sensors. Moreover, the continuum lattice body provides adequate stiffness to support these devices and other payloads. Finally, the soft hollow body allows for adaptability to deformations of flexible pipelines. These properties have been validated through our experiments. Furthermore, we demonstrate two potential applications of the designed robot in obstructed concealed pipelines and disturbed offshore pipelines, which are challenges that conventional designs struggle to address.

## 2 Results

### 2.1 Design of Shell‐Lattice Soft Robots

**Figure 2:**
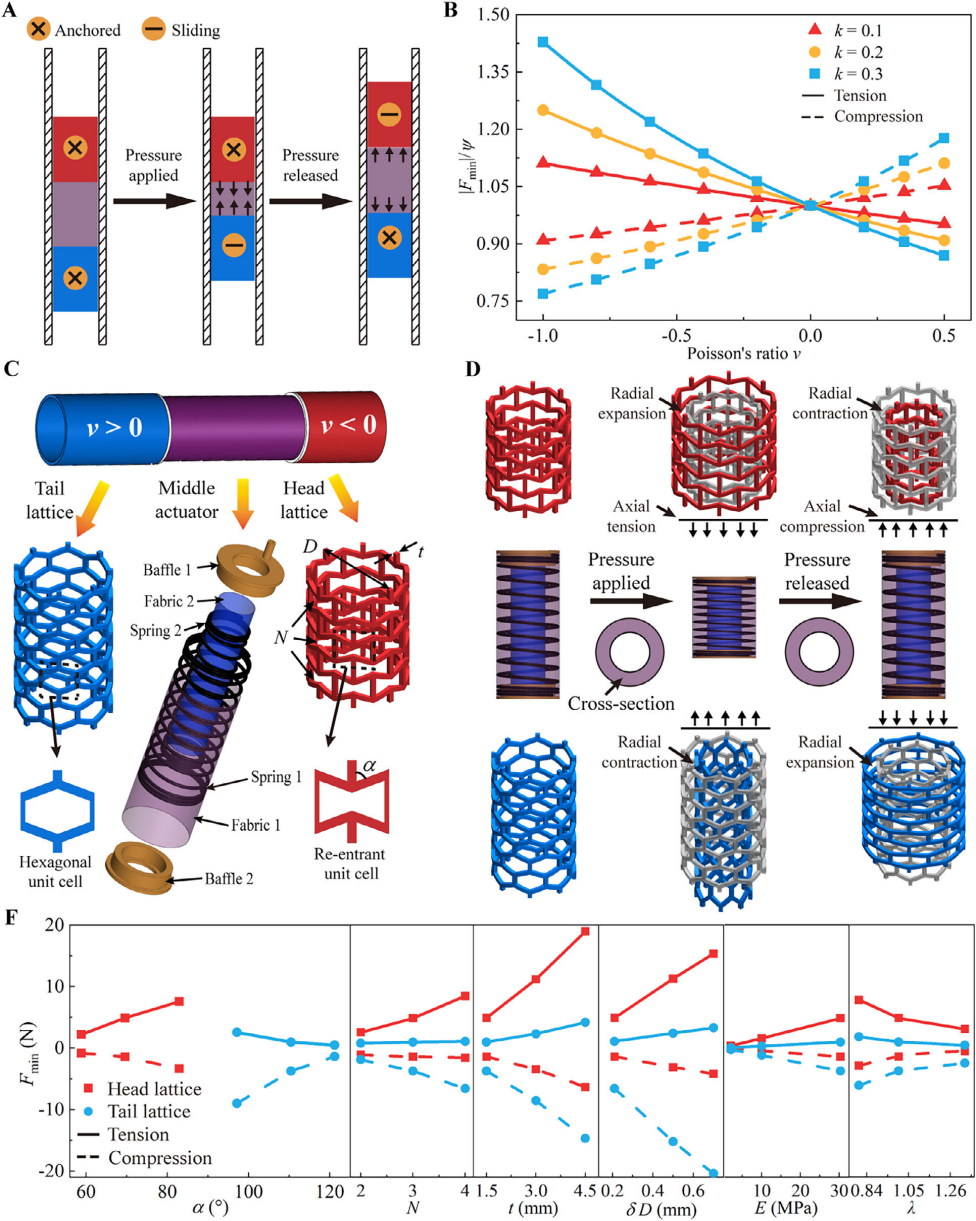
Design and locomotion mechanism of shell‐lattice soft crawling robots. A) Schematic illustration of the earthworm‐like locomotion mode of the proposed robot. B) Variation of the normalized minimum sliding force |*F*_min_|/*Ψ* with Poisson's ratios of the soft materials when the cylindrical shells are compressed and stretched within a pipe. C) Design diagram by employing lattice shells instead of cylindrical shells for pursuing desired effective Poisson's ratios, as well as the exploded view of the middle actuator. D) Deformation mechanisms of the (top) head lattice, (middle) actuator, and (bottom) tail lattice under applied and released pressures. The gray and color regions indicate un‐deformed and deformed states, respectively. E) Variation of *F*_min_ with four parameters representing the geometries of the lattice cells, material properties, and unit cell size factors.

Our designed robot consists of three hollow cylindrical sections, including a pneumatic actuator in the middle and two passive cylindrical shells as the head and tail sections (Figures 1 and **2**A). Negative air pressures are repeatedly applied and released to the actuator, generating axially tensile and compressive forces to the two cylindrical shells, respectively. In each loading scenario, both shell sections maintain tight contact with the inner wall of the pipe, but they induce different frictional forces to facilitate crawling. During each actuation cycle, the head section is deformed to anchor itself when negative pressure is applied, while the tail section is dragged forward. Conversely, when the pressure is released, the tail section anchors while the head section moves forward (**Figure** 2A). This cyclical loading and unloading process enables the robot to crawl in an earthworm‐like manner.

The middle actuator consists of two springs with different diameters, two end baffles, and two side fabrics, collectively forming a closed air chamber (Figure 2C). It is connected to an external pump via an air tube, which provides air pressure. During actuation, as negative air pressure is applied to the chamber, the two end baffles experience an inward pressure directed toward the chamber. This pressure causes the springs to be compressed, resulting in contraction of the actuator. Consequently, the actuator generates axial tension that acts on both the head and tail cylindrical shells. Given that the springs have significantly higher radial stiffness compared to its axial stiffness, they undergo negligible radial deformation. Therefore, the cross section of the actuator remains nearly constant throughout the actuation process (Figure 2D). In contrast, when the applied negative pressure is released, the compressed springs expand, allowing the actuator to recover its length. At this stage, the actuator exerts axial compression on both cylindrical shells. Applying negative pressures prevents the blockage of the hollow area and excessive contact with the pipe walls, which could occur under positive pressure. Moreover, the embedded springs mitigate the risk of sealed chambers caused by excessive radial shrinkage of the side fabrics during increased negative pressures.

The design of the head and tail cylindrical shells is critical for achieving the proposed periodic earthworm‐like locomotion. We have derived several design principles based on the analysis of the minimal axial forces required for sliding, denoted by the minimal sliding force *F*_min_, which accounts for the friction between the head/tail sections and the inner wall of the pipe. Specifically, if one shell exhibits a smaller *F*_min_ than the other under compressive loading, it must correspondingly have a larger *F*_min_ under tensile loading. This design ensures that, regardless of whether a compressive or tensile force is exerted by the pneumatic actuator, only one of the two cylindrical sections remains anchored while the other slides. In addition, it is preferable to design the lattice shell sections with a sufficiently large difference in *F*_min_. This approach significantly enhances the robustness of the aforementioned relationship in *F*_min_, even in the presence of geometric deviations or rough surfaces due to manufacturing defects. Moreover, the difference in *F*_min_ between the two shells determines the maximum payload that the robot can carry (Note S4, Supporting Information). Finally, smaller *F*_min_ values that adhere to these fundamental design principles are preferred, as they facilitate greater elastic energy storage in the compressed springs and enable longer movement distances.

According to the elasticity theory and force balance law, the minimal force *F*_min_ to slide an individual cylindrical shell is expressed by

(1) $$F_{\min}=\left\{\begin{array}{c} \frac{\psi }{1+kv}\;\mathrm{for}\;\mathrm{tensions}\\ -\frac{\psi }{1-kv}\;\mathrm{for}\;\mathrm{compressions} \end{array}\right.$$

where *Ψ* = *2πµEthδD*/(*D‐t*) and *k* = *2µh*/(*D‐t*). Here, *D*, *t*, and *h* are the outer diameter, the wall thickness, and the height of the cylindrical shell (Figure S2, Supporting Information), respectively; *δD* is the interference amount between the cylindrical shell and the pipe; *E* and *v* are Young's modulus and Poisson's ratio of the soft constituent material, respectively; and *µ* is the frictional coefficient between the cylindrical shells and the pipe. The readers are referred to Note S2 (Supporting Information) for details about the derivation of Equation (1).

It is observed that the Poisson's ratio *v* of the soft constituent material has a dominant influence on *F*_min_ (Figure 2B), because it determines the radial deformations of the cylindrical shells and consequently the normal contact pressure between the shell structures and the pipe wall under axial loading. When subjected to axial tension, a cylindrical shell with *v* > 0 has a smaller *F*_min_ due to radial contraction, whereas a shell with *v* < 0 exhibits a greater *F*_min_ owing to its radial expansion. Conversely, under axial compression, a cylindrical shell with *v* > 0 has a greater *F*_min_, while one with *v* < 0 has a smaller *F*_min_.

To maximize the absolute difference of *F*_min_ between two sections, we require the head and tail shells to possess different signs of their Poisson's ratios *v*. Specifically, we implement a configuration where the head shell has *v* < 0 and the tail shell has *v* > 0. In this design, when negative pressure is applied to the actuator, the head cylindrical shell undergoes radial expansion and anchors to the pipe, while the tail shell contracts away from the pipe. Once *F*_min_ for the tail section is reached, the tail cylindrical shell is dragged forward. Upon removal of the pressure, the tail cylindrical shell anchors, while the head shell contracts and is subsequently pushed forward once *F*_min_ for the head section is reached. In this way, the expected earthworm‐like locomotion is reproduced.

However, soft materials with the desired negative Poisson's ratios are rarely found in nature. Instead, soft lattice structures can be engineered to achieve specific effective Poisson's ratios over the theoretical bound.^[^ ^39^, ^40^, ^41^, ^42^, ^43^, ^44^, ^45^ ^]^ Therefore, we employ periodically‐arrayed lattice structures to replace the cylindrical shells, where the re‐entrant and hexagonal unit cells are used for design of the head and tail lattice shells, respectively (Figure 2C; Figure S3, Supporting Information). Each type of unit cell is characterized by an angle *α* between the inclined and vertical struts. The re‐entrant unit cells are characterized by 0° < *α* < 90°, and the hexagonal unit cells fall within the range 90° < *α* < 180°. In addition, the geometries of the lattice shells are represented by three parameters, the number of unit cells along the axis *N*, the wall thickness *t*, and the outer diameter of the cylinder *D*.

The deformation mechanisms of the two lattice shells are governed by the configurations of their unit cells. In the case of re‐entrant configuration, each unit cell contracts horizontally as vertical compression is applied, since *α* between the vertical and inclined struts decreases (Figure S4A, Supporting Information). Conversely, the unit cell expands horizontally under vertical stretching. Consequently, the lattice shell composed of periodic re‐entrant unit cells undergoes radial expansion and contraction under uniaxial tension and compression (Figure 2D), respectively. In contrast, the single hexagonal unit cells expand and contract in the horizontal direction under vertical compression and tension (Figure S4B, Supporting Information), respectively. Therefore, the tail lattice shell undergoes radial deformations that are opposite to those of the head lattice shell when subjected to the same uniaxial loading (Figure 2D).

A numerical parameter study is conducted to investigate the influence of six parameters on the minimal sliding force *F*_min_. The values of these parameters are prescribed in Table S1 (Supporting Information), and the comparison results are illustrated in Figure 2E. First, both types of lattice shells approaching *α* = 90° obtain larger *F*_min_. This can be attributed to their enhanced hoop stiffness,^[^ ^46^ ^]^ resulting in increased normal contact forces against the pipe wall (Figure S5, Supporting Information). Second, increasing either the lattice shell thickness *t*, or the number of unit cells along the axis *N*, yields larger magnitudes of *F*_min_. This is because thicker lattices possess enhanced stiffness, while an increased number of unit cells enlarges the contact area. Third, a larger initial interference amount *δD* also contributes to higher *F*_min_, due to tighter contact between the lattice bodies and the pipe wall. It is worth noting that the minimal sliding forces of the head and tail lattices satisfy the design principals across a broad range of *δD*. This indicates that the robot can crawl in the pipes even amid manufacturing variations, thus demonstrating the robustness of the design. Fourth, the stiffness of the base material also influences *F*_min_. When the pipe material is fixed, a higher stiffness of the base material leads to a larger *F*_min_ due to the enhanced hoop stiffness of the lattice shell. It is noted that using a material that is too stiff reduces the adaptability of the robot to curved pipes, while a material that is too soft results in poor load‐bearing capability. Lastly, a scaling factor *λ* is introduced to adjust the cell size, where a larger *λ* corresponds to a larger unit cell. Adjusting *λ* alters the contact area between the robot and the pipe wall, thereby affecting *F*_min_. In general, smaller unit cells lead to larger contact areas as more unit cells are included, leading to larger *F*_min_. More details about simulations can be found in the Experimental Section.

### 2.2 Fabrication and Characterization of Actuator and Lattice Shells

Several specimens of the designed shell‐lattice soft robot are fabricated to validate their crawling capabilities. The head and tail lattices consist of *N* = 3 and *N* = 4 unit cells along the axis, with *α =* 69.54° and *α =* 110.46°, respectively. Each unit cell measures 12.06 mm in width and 13.5 mm in height. Both lattice shells feature a wall thickness of 1.5 mm and an outer diameter of 32.21 mm. Based on the numerical results presented in Figure 2E, such a design satisfies all the aforementioned design principals. Specifically, among all potential designs, employment of *N* = 3 and *N* = 4 unit cells yields the largest minimum difference in *F*_min_ between the head and tail lattices under tension and compression, which ensures optimal load‐bearing capability (Note S4, Supporting Information). The actuator has an inner diameter of 16.00 mm, an outer diameter of 29.00 mm, and a height of 71.00 mm. The total compressive stiffness of the two embedded springs is measured at 1.15 N mm^−1^. Each lattice shell is 3D printed as an integrated component, while the hollow actuator is made through an assembly process. The shell‐lattice robot is assembled by bonding three components together (Movie S1 and Figure S6, Supporting Information).

**Figure 3:**
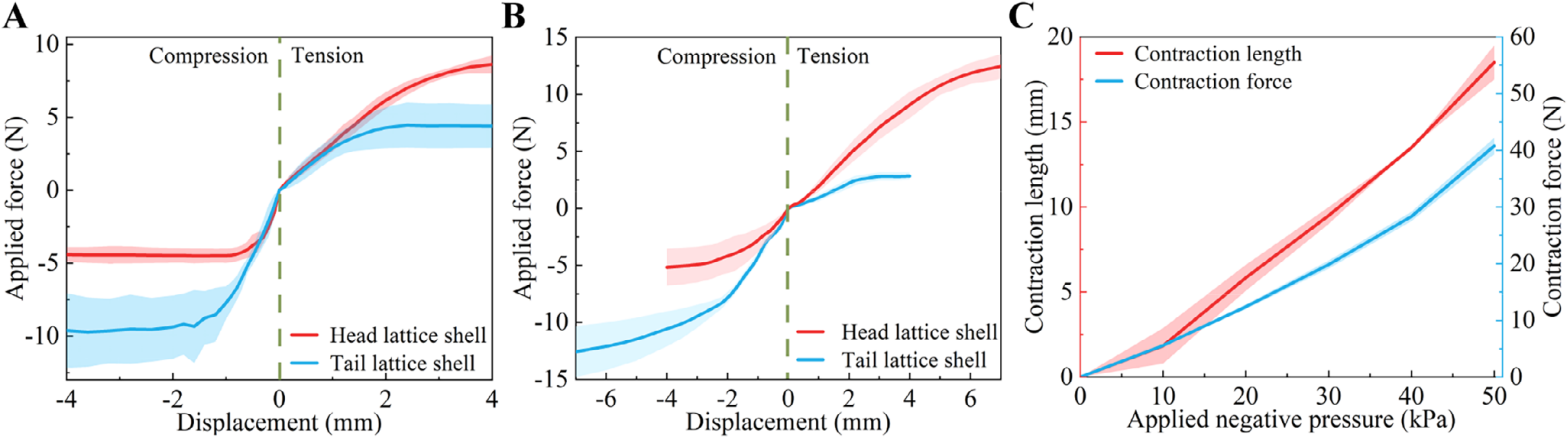
Characterization of the lattice shells and the actuator. Force‐displacement curves under an applied load enabling sliding of the lattice shells A) in a dry glass pipe and B) in an underwater pipe. C) Contraction length and contraction force of the actuator under different pressures. In both subplots, the solid lines represent the averaged responses of three samples, while the transparent bands denote the standard deviation measured in experiments.

To characterize *F*_min_ of the 3D‐printed head and tail lattice shells, we place them into a dry glass pipe and an underwater pipe, respectively, and axially drag or push them from one end (Figure S7A,B, Supporting Information). The pipe has an inner diameter of 32.00 mm. Initially, the applied force increases with the local axial deformation at the loading end, while the entire lattice body remains stationary (**Figure** **3**A,B). Once the loaded lattice section begins to slide, the applied force stabilizes and this value is recorded as *F*_min_. For axially tensile and compressive loadings in the dry glass pipe, the measured average values of *F*_min_ for the head lattice are 8.79 N and −4.42 N, and those for the tail lattice are 4.37 N and −9.45 N, respectively. When the glass pipe is submerged underwater, the measured average value of *F*_min_ for the head lattice is 5.17 N under uniaxial compression (Figure 3B), while it increases to 12.43 N under uniaxial tension. For the tail lattice, the values of *F*_min_ are 2.83 N and 12.57 N under tension and compression, respectively. These test results validate the effectiveness of the lattice shell design and the design principles outlined above.

Furthermore, the contraction performance of the actuator is also characterized through experiments (Figure S7C, Supporting Information). When an air pressure of ‐50 kPa is applied, the actuator exhibits a measured contraction force of 40.78 N and a contraction length of 18.50 mm (Figure 3C). It is evident that the contraction force significantly exceeds the combined minimal sliding forces *F*_min_ of the head and tail lattice shell sections. Additionally, the maximum contraction length exceeds the local displacement at the loading end of the lattice shells before sliding. As a result, the actuator is capable of generating sufficient forces to initiate the sliding of the lattice shells, thereby facilitating the crawling motion of the robot. More details about the fabrication process, the experimental setups, and the measurement methods can be found in the Experimental Section.

### 2.3 Capabilities of Shell‐Lattice Soft Robot

#### 2.3.1 Crawling in Pipes with Flowing Fluids

**Figure 4:**
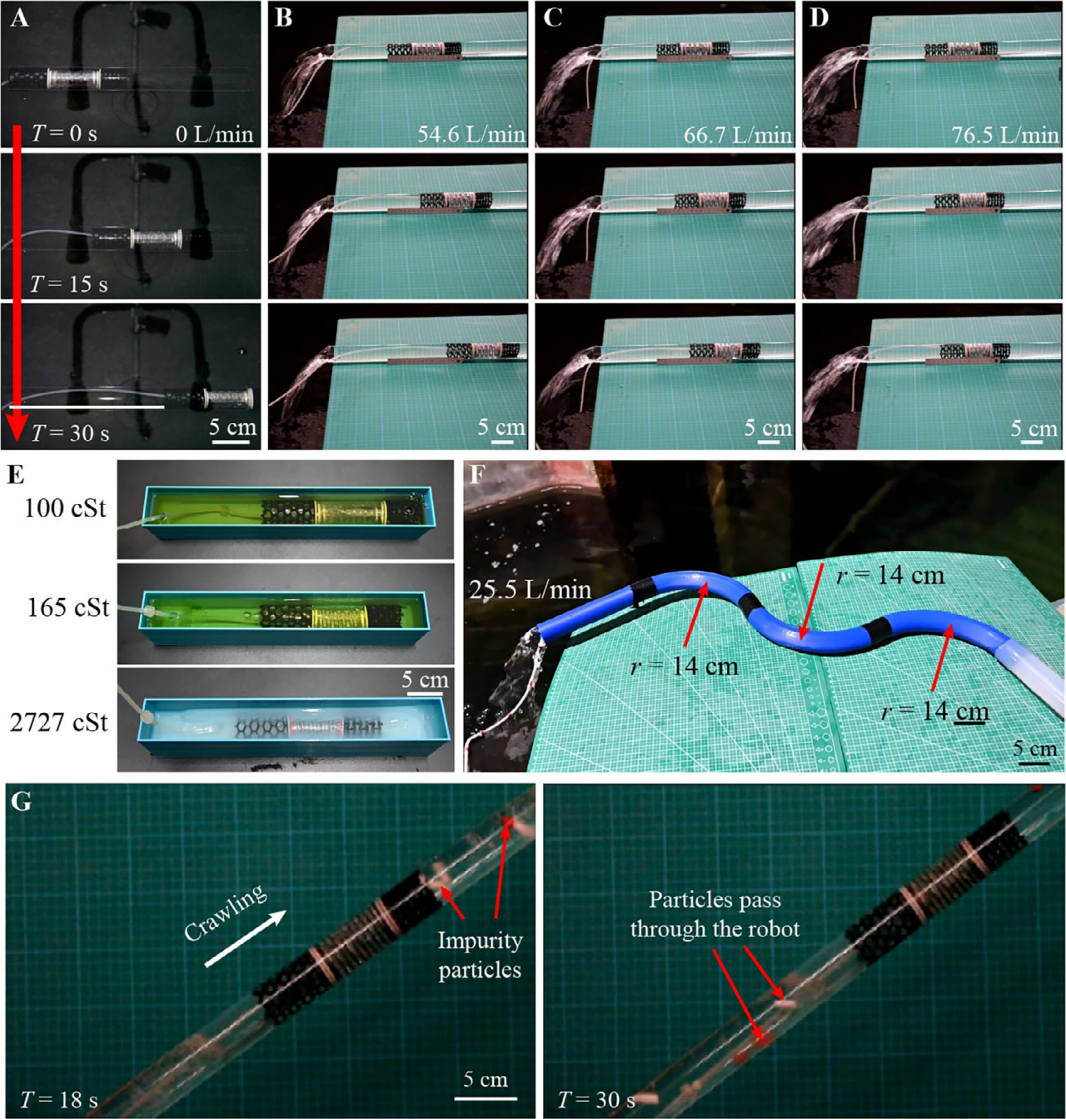
Crawling in fluid pipes. Crawling in glass pipes with flowing water, with the flow rates of A) 0 L min^−1^, B) 54.6 L min^−1^, C) 66.7 L min^−1^, and D) 76.5 L min^−1^. E) Crawling in pipes filled with fluids of varying viscosities. F) Crawling in doubly‐curved rigid pipe with higher‐velocity fluids. G) Crawling in a pipe with flowing fluid mixed with impurity particles.

The most critical feature of the designed robot is its ability to facilitate the normal transport of flowing fluids while crawling within a pipe. For verification, the robot is placed in a glass pipe with flowing water (Note S3, Supporting Information), where the flow rate varies from 0 L min^−1^ for still water to a maximum of 76.5 L min^−1^ for flushing water. In the latter scenario, the average flow velocity of the fluid exceeds 1.50 m s^−1^, with a high Reynolds number exceeding 50 000, which is a typical speed in water supply networks.^[^ ^47^ ^]^ The testing results confirm that the robot can effectively crawl against flowing water (**Figure** **4**A–D; Movie S2, Supporting Information). This performance is primarily attributed to the smooth passage of water through the hollow regions of the robot. Moreover, the thin‐walled shell structure minimizes the fluid resistance. The surface‐to‐surface contact between the lattice bodies and the pipe provides sufficient friction, therefore ensuring a stable anchoring of the lattice bodies against flushing flows. According to the simulation results (Figure S1C, Supporting Information), the drag force increases with flow velocity. When crawling in water, the drag force on the robot increases from 0.07 to 16.46 N as the flow velocity varies from 0.1 to 1.59 m s^−1^ (Note S1, Supporting Information). A more significant increase can be observed when crawling in fluids with higher viscosities. Due to this rise in drag force, the crawling speed of the robot decreases from 7.03 mm s^−1^ in still water to 1.20 mm s^−1^ in flushing water (Figure 4D).

The crawling performance of the robot in pipes filled with various fluids has also been tested (Figure 4E). Three types of fluids are used, including oil,  ultra‐tough liquid (UTL) resin solution, and uncured silicone rubber Ecoflex 0030, with viscosities of 100 cSt, 165 cSt, and 2727 cSt, respectively. The experimental results demonstrate that the robot successfully crawls through the pipes containing these fluids (Movie S2, Supporting Information). A comparison of the crawling performance reveals a decrease in the crawling speed from 12.5 to 4.41 mm s^−1^ as the viscosity increases from 100 to 2727 cSt. This indicates that the robot experiences greater drag forces from the fluids with higher viscosities, which is also confirmed by the simulation results (Figure S1C, Supporting Information). The frictional forces exerted by the fluids on the side faces of the robot account for 10–30% of the drag forces (Figure S1C,D, Supporting Information). As the fluid viscosity increases, the proportion of the fluid frictional forces also increases, thereby affecting the crawling speed of the robot. Both experimental and simulation results indicate that the proposed robot can adapt to a wide range of fluids. While both higher fluid velocity and viscosity increase the drag forces acting on the robot, the effect of the fluid velocity is more pronounced. For instance, the drag force in a fluid with a viscosity of 2727 cSt and a velocity of 0.1 m s^−1^ is 10.61 N, which is lower than the drag force of 16.46 N encountered in a fluid with a viscosity of 1.01 cSt and a velocity of 1.585 m s^−1^. This suggests that the robot can adapt to high‐viscosity but low‐velocity liquids.

The crawling capability of the robot in complex fluid environments has been further evaluated. A 3D‐printed double‐curved serpentine pipe is used for robot crawling (Figure 4F), with the pipe filled by flushing water. Specifically, two scenarios are tested. In the first scenario, high‐velocity water is supplied, with a maximum fluid flow rate of 25.5 L min^−1^, corresponding to a fluid velocity of 0.5 m s^−1^ and a Reynolds number exceeding 15 000. Such a velocity typically generates turbulent flow, and even fluid vortexes and vorticity around the curved arcs within the pipe. In the second scenario, water with a periodic variation in flow rates between 8.5 L min^−1^ and 24.2 L min^−1^ is supplied, producing wave propagation. Despite these challenging working conditions, the robot successfully demonstrates its ability to crawl against the flushing flow (Movie S2, Supporting Information). Particularly, the robot smoothly crawls through the arcs, without being disturbed by the complex fluid environments. These results highlight the strong adaptability of the robot to diverse fluid conditions.

Moreover, the hollow shell design allows the robot to move through pipes containing flowing fluids mixed with particulate impurities. Two groups of polylactic acid (PLA) particles are 3D printed (Figure 4G; Figure S8, Supporting Information). One group consists of five types of polyhedron particles, with a maximum size of ≈15 mm, which is roughly equal to the diameter of the hollow section of the robot. The other group includes hemisphere particles with a diameter of 10.00 mm, as well as cylindrical particles with a diameter of 6.00 mm and a height of 20.00 mm. These particles are mixed with flowing fluids. Experimental results show that all the polyhedron particles traverse smoothly through the crawling robot, without obstructing its movement or causing blockages (Figure S8; Movie S2, Supporting Information). Interestingly, even the cylindrical particles have larger height than the robot section, they can pass through the crawling robot without causing blockage (Figure 4G; Movie S2, Supporting Information). This capability is highly remarked to enhance the suitability and adaptability of our robot for various applications compared to other existing soft robots, which occupy the cross‐sections of the pipes.^[^ ^29^, ^30^, ^34^ ^]^

#### 2.3.2 Crawling in Various Types of Pipes

**Figure 5:**
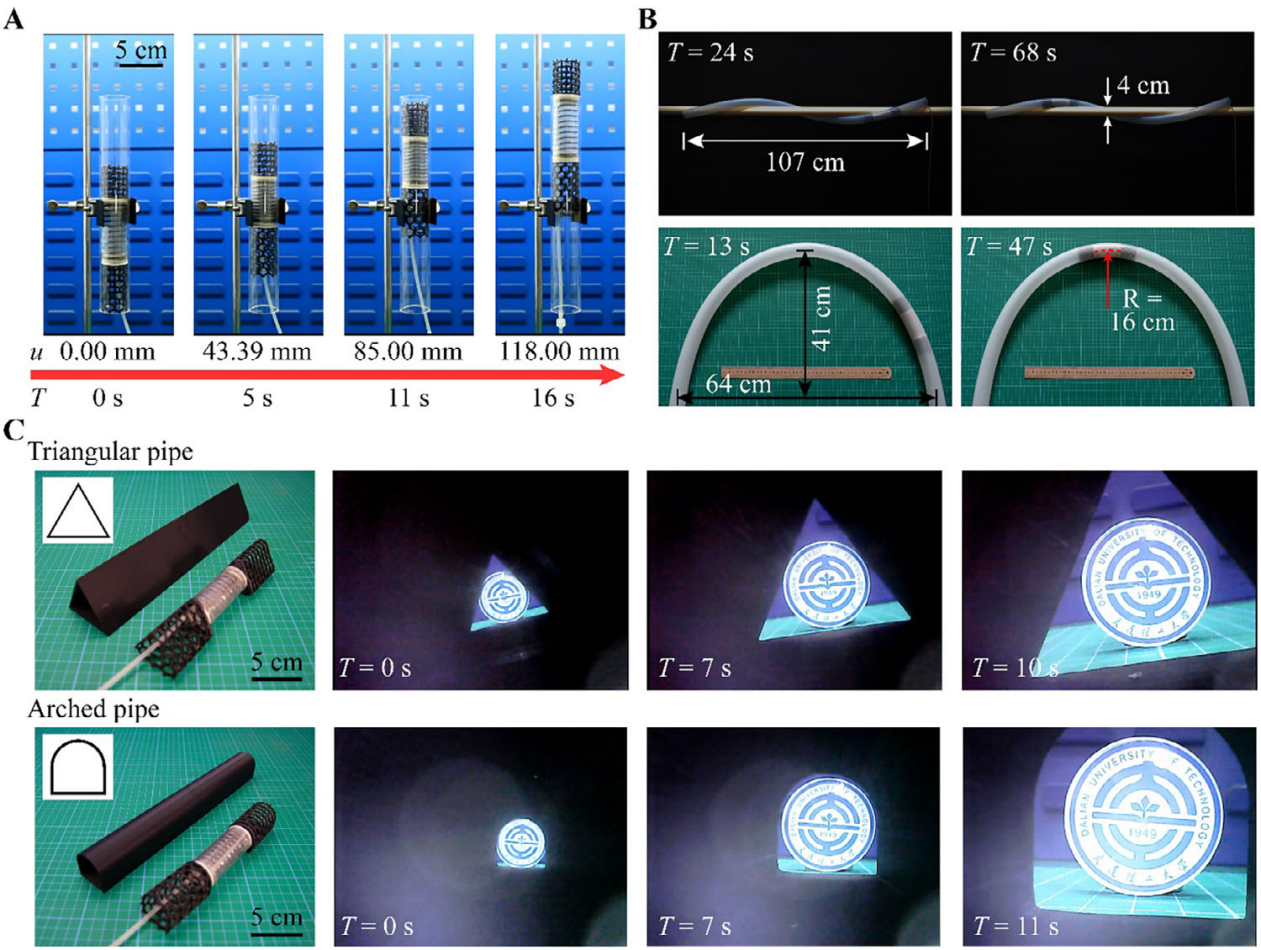
Crawling in various pipes. A) Crawling in a dry glass pipe. B) Crawling in curved silicone pipes. C) Crawling in pipes with triangular and arched cross‐sections.

Next, we evaluate the crawling capability of our robot in various pipe types. First, the robot is placed in a series of pipes with different frictional coefficients between the robot and the pipe walls. Five pipes are used, including three dry pipes made from glass, 304 stainless steel and acrylic, as well as two glass pipes with their inner walls coated in water and vacuum pump oil. The frictional coefficients between the lattice shells and the pipes range from 0.25 to 0.55 (Figure S9, Supporting Information). For pipes made from materials with higher frictional coefficients, such as acrylic, larger *F*_min_ values are typically required to move the robot, as indicated by Equation (1). This in turn affects the maximum moving distance and the crawling speed of the robot within a single actuation period. It is noted that variation of the frictional coefficients only impacts the magnitudes of *F*_min_, while the relative relationship between the *F*_min_ values of the head and tail lattices remains consistent. This ensures that the robot can effectively adapt to the pipes made from various materials or coated with different substances that exhibit varying frictional coefficients. The test results demonstrate that the robot is able to crawl from the bottom to the top in all these pipes (**Figure** **5**A; Movie S3, Supporting Information). It is worth noting that the frictional coefficients can also be adjusted by coating the robot surfaces with specific materials or by incorporating custom‐designed textured surfaces.^[^ ^19^, ^38^ ^]^ In general, the lattice shells with more complex textures or greater roughness lead to higher frictional coefficients, thereby generating higher *F*_min_ values against the pipe wall. To date, various methods have been proposed to design the textures on lattice surfaces.^[^ ^48^, ^49^ ^]^ This surface engineering technology could enhance the adaptability of our robot for a wide range of scenarios.

Second, the crawling capability of the robot within curved pipes is validated. A silicone pipe with a wall thickness of 3.0 mm and a section diameter of 35 mm is bent into a 3D helical shape and a planar U‐shape with a minimum curvature radius at the top of ≈16.00 cm. The soft lattice body imparts a relatively low bending stiffness, enabling the robot to conform to various spatial curvatures and to crawl through these curved pipes (Figure 5B; Movie S3, Supporting Information). In the case of the planar U‐shaped pipe, the bending angle between the head and tail ends can reach up to 22°. Notably, the robot is capable of adapting to the varying cross‐sectional shape of the flexible pipe, which deforms to a non‐circular geometry in the bent state. This capability indicates the excellent adaptability of the robot to flexible pipes that deform under external forces, such as flexible pipelines in oceans subjected to waves and currents.

To assess the minimum local curvature that the robot can crawl through, its performance is tested in crawling through five 3D‐printed U‐shaped rigid pipes with curvature radii ranging from 80 to 160 mm (Figure S10, Supporting Information). These pipes are made from PLA, which has a Young's modulus ≈100 times higher than that of the shell‐lattice robot. The experimental results demonstrate that the robot successfully crawls through the pipes with local curvature radii greater than 100 mm (Movie S3, Supporting Information). For the pipe of curvature radius 100 mm, the head lattice turns ≈100°. The minimum local curvature radius is influenced by both the section size of the pipe and the dimensions of the robot.

Finally, two additional shell‐lattice robot bodies are 3D printed, which is composed of the same unit cells as those in Figure 2C but featuring triangular and arched cross‐sectional shapes (Figure S11A,B, Supporting Information). The corresponding robots are assembled by attaching them to the middle actuator (Experimental Section). It is worth noting that these lattice bodies exhibit similar radial deformation modes as those with circular cross‐sections, as radial deformations are primarily dictated by the unit cells used. Moreover, the measured *F*_min_ of the head and tail lattices meet our design principals, allowing the robots to crawl effectively in pipes with non‐circular cross‐sections (Figure 5C; Figure S11C,D and Movie S3, Supporting Information). These customized lattice bodies enhance the versatility of the designed robots for various pipeline scenarios of working space restrictions, installation constraints, and functional requirements.^[^ ^50^, ^51^ ^]^

#### 2.3.3 Crawling with a Payload

**Figure 6:**
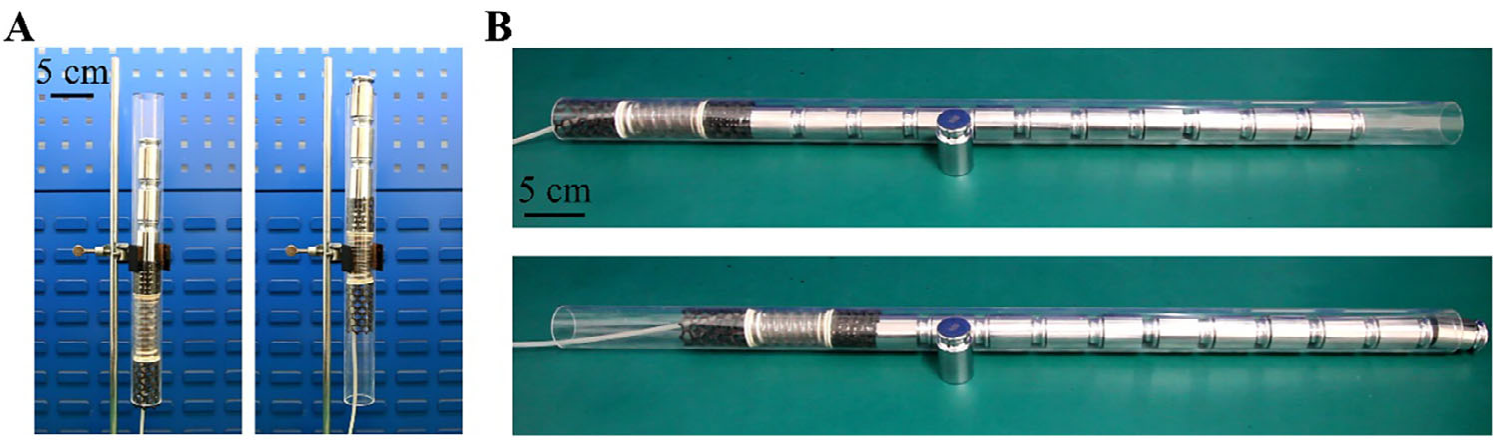
Crawling of the robot in glass pipes with a payload. A) Crawling vertically with a payload of 700 g. B) Crawling horizontally with a payload of 2300 g.

The load‐bearing capacity of the shell‐lattice soft robot is further validated, which is a critical factor for transporting functional devices necessary for inspection and cleaning tasks. In our experiments, the payload is positioned on the connecting plate at the end of the head section (Figure S12A, Supporting Information). Despite the weight of only 45.09 g, the robot successfully carries payloads of 700 g and 2300 g while crawling in vertical and horizontal pipes, respectively (**Figure** **6**; Movie S4, Supporting Information). These payloads are more than 15 and 50 times the robot weight, indicating its excellent load‐bearing capability. This strength is attributed to the substantial difference in the minimal sliding forces between the head and tail sections (Note S4, Supporting Information), which not only ensures crawling stability but also enables the robot to transport devices for pipeline inspection and maintenance during operation.

#### 2.3.4 Untethered Crawling

**Figure 7:**
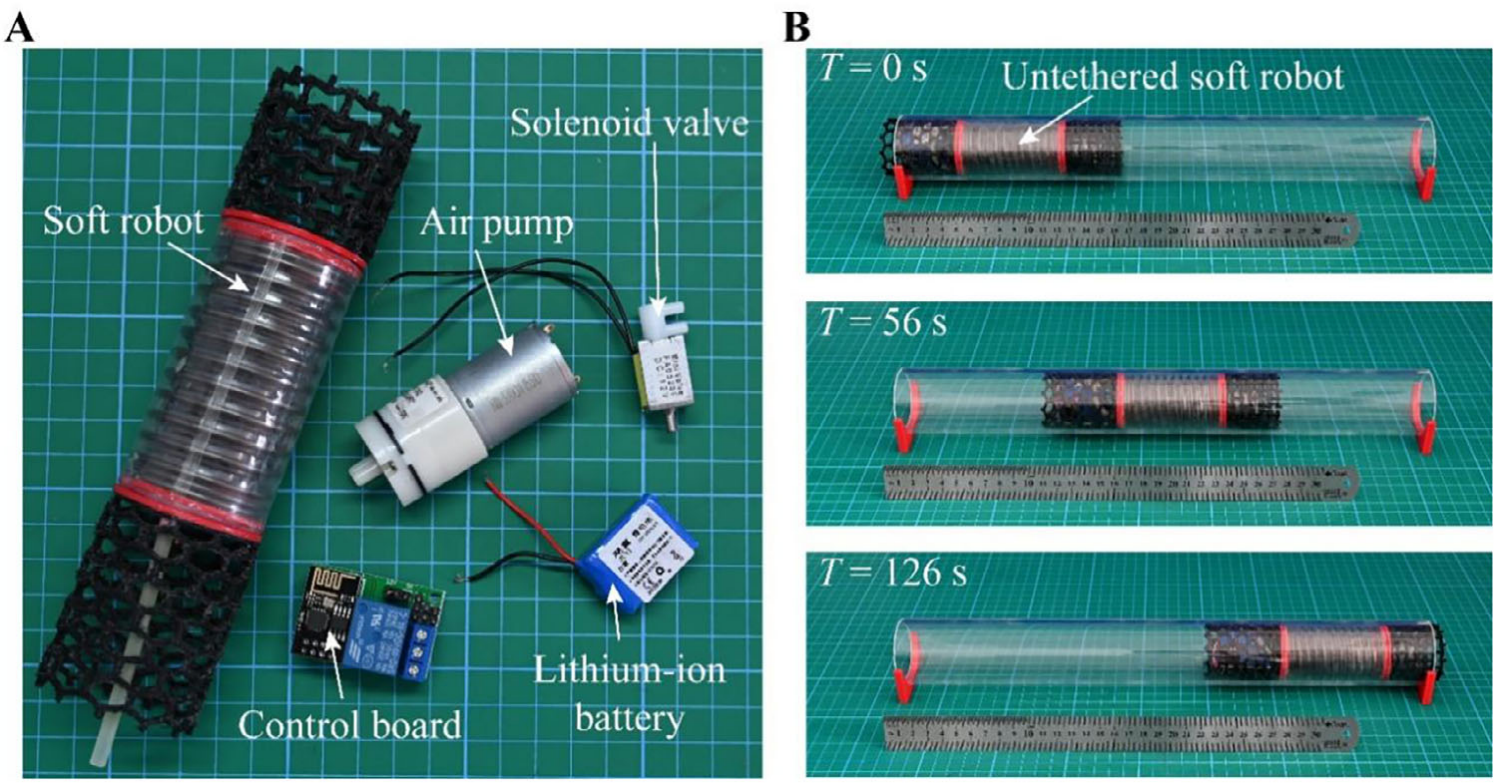
Untethered shell‐lattice soft crawling robot. A) Untethered design. B) Untethered crawling.

Finally, our robot demonstrates the capability for untethered locomotion. Typically, the maximum crawling distance of a soft pneumatic robot is restricted by the length of the trailing tube connected to an air pump for actuation. This limitation poses a significant challenge for pipelines that extend hundreds or even thousands of meters, such as those used in gas and oil transportation. To address this issue, we have integrated a micro air pump and complementary electronic devices within the hollow space of the actuator section (Experimental Section; Figure S14, Supporting Information). This configuration allows the robot to autonomously generate and regulate periodic air pressures to enable crawling. As a result, the untethered robot can crawl through a glass pipe without the need for a trailing tube (**Figure** **7**; Movie S5, Supporting Information). Under the current configuration, the robot achieves a crawling speed of 1.96 mm s^−1^. The endurance time and maximum crawling distance are determined by the battery capacity (Note S5, Supporting Information). As equipped with a 12 V, 150 mAh battery, the robot can travel up to 5.08 m. Using this untethered design for crawling in water‐filled pipes may require an additional airbag to store the air expelled from the actuator. Moreover, it needs a powerful pump to transfer air between the actuator and the airbag against water pressure.

#### 2.3.5 Comparison of Crawling Performance with Existing Robots

The proposed pipeline‐crawling robot is compared with two groups of existing robot designs, including five representative pneumatic soft robots,^[^ ^29^, ^31^, ^33^, ^34^, ^36^ ^]^ and three magnetically‐driven soft robots,^[^ ^3^, ^23^, ^38^ ^]^ in terms of structural characteristics, motion modes, actuators, crawling speeds in pipes with air and fluids, load‐bearing capability, ability to crawl in flowing fluids, and fluidic drag forces. A detailed comparison table is provided in Table S2 (Supporting Information).

Within the category of pneumatic soft robots, most existing designs feature block‐like bodies with enclosed air chambers.^[^ ^29^, ^31^, ^33^, ^34^ ^]^ These robots encounter significant challenges when crawling through pipes filled with flowing fluids, as they experience substantial drag forces and potential obstruction from impurities. To achieve earthworm‐like motion, these designs typically require multiple actuators. In contrast, the hollow body of the proposed robot enables it to crawl efficiently through pipes containing high‐velocity flowing fluids, even those mixed with impurities. Additionally, the robot employs only one actuator to achieve higher load‐bearing capability than the other existing pneumatic robot counterparts. According to Table S2 (Supporting Information), the crawling speed of the proposed robot in air‐filled pipes surpasses that of most existing pneumatic robots,^[^ ^29^, ^33^ ^]^ though it is slower than the fastest design.^[^ ^36^ ^]^ As the crawling speeds are affected by factors such as the dimensions and material properties of both the pipes and robot, as well as actuation frequency, a fair comparison typically requires identical testing conditions.

Magnetically‐driven soft robots are generally characterized by small microfiber,^[^ ^3^ ^]^ thin sheet‐shaped,^[^ ^23^ ^]^ or stent‐like^[^ ^38^ ^]^ architectures. These robots feature hollow bodies, allowing them to crawl through fluids, and are commonly used for health monitoring in biological pipelines with relatively low Reynolds numbers. They primarily utilize rotational motion for crawling,^[^ ^3^, ^38^ ^]^ relying on non‐uniform frictional forces against the pipe walls. Due to their tiny sizes, it is a challenge for these robots to take heavy objects during crawling. In comparison, the proposed robot excels in crawling through complex fluid environments, including those with higher Reynolds numbers, higher flow velocities, and wave‐like fluid dynamics. Moreover, its large contact area with pipe walls enhances its load‐bearing capability. Since the proposed robot is relatively larger than the magnetically‐driven robots, it achieves higher crawling speeds but comes with increased drag forces.

### 2.4 Potential Applications of Shell‐Lattice Soft Crawling Robot

#### 2.4.1 Pipeline Inspection and Obstacle Cleaning in Concealed Pipelines

The first potential application scenario for our designed robot involves the inspection and maintenance of concealed pipelines. These pipelines, including underground pipelines for water supply, heating, and energy transport, frequently encounter issues such as blockages and leaks, and therefore, continuous monitoring is necessary to maintain their operational integrity. However, the internal environments of these pipes are difficult to observe, and high fluid pressures restrict the effectiveness of existing in‐pipe soft robots that occupy the cross‐sections. Moreover, harsh underground conditions pose considerable challenges for out‐of‐pipe robots, hindering effective inspection, maintenance, and cleaning of obstacles within the pipes.

**Figure 8:**
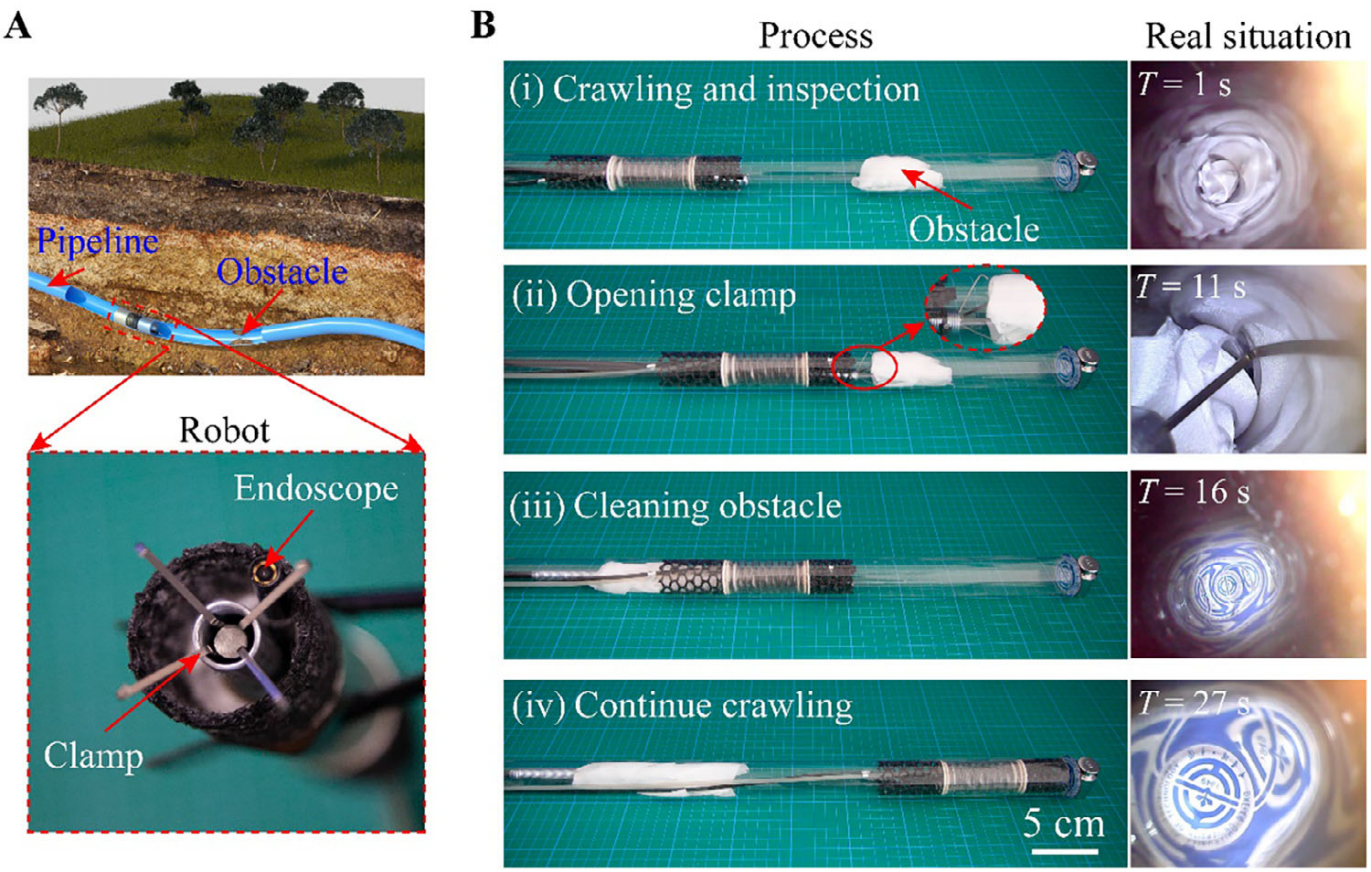
Shell‐lattice soft crawling robot designed for inspecting and cleaning concealed pipelines. A) Illustration of a pipeline with obstacles and the soft robot equipped with obstacle‐cleaning devices. B) Cleaning of the pipe blocked with a cloth and the real view from the endoscope.

Our designed shell‐lattice robot presents an effective solution to these challenges, as demonstrated through experiments with a test robot. Specifically, it houses a 5.5 mm diameter high‐resolution endoscope (HD300, Shenzhen Dannan Technology Co., Ltd.), mounted on the head section and connected to a computer via a wire. It also features a cleaning clamp designed to remove obstacles encountered within the pipes. To simulate an obscured and obstructed pipe environment (**Figure** **8**A), a glass pipe is covered with an opaque film, and a piece of cloth is placed inside to block the passage (Figure 8B). An insignia is positioned at the pipe end to test the cleaning efficacy.

In our experiments, the proposed robot demonstrates robust performance in conducting inspection and cleaning tasks within the pipe (Figure 8B; Movie S6, Supporting Information). The real‐time transmission of captured images from the integrated endoscope enables live monitoring and accurate location of blockages. During crawling, the robot maneuvers to a designated position near the obstruction, allowing the operator to conduct interior inspections and remove obstacles using a tool passing through the hollow channel of the robot. Subsequently, the robot resumes crawling to carry out further inspection tasks, culminating in the complete visualization of the insignia at the pipe end through the endoscope. This process exemplifies the remote‐controlled ability of the robot to identify and clean pipelines using a visual identity system, highlighting its potential for pipeline maintenance operations. In addition, the functional devices can be operated through both wired and wireless modes, depending on the working requirements.

#### 2.4.2 Crawling in Flexible Ocean Pipelines

**Figure 9:**
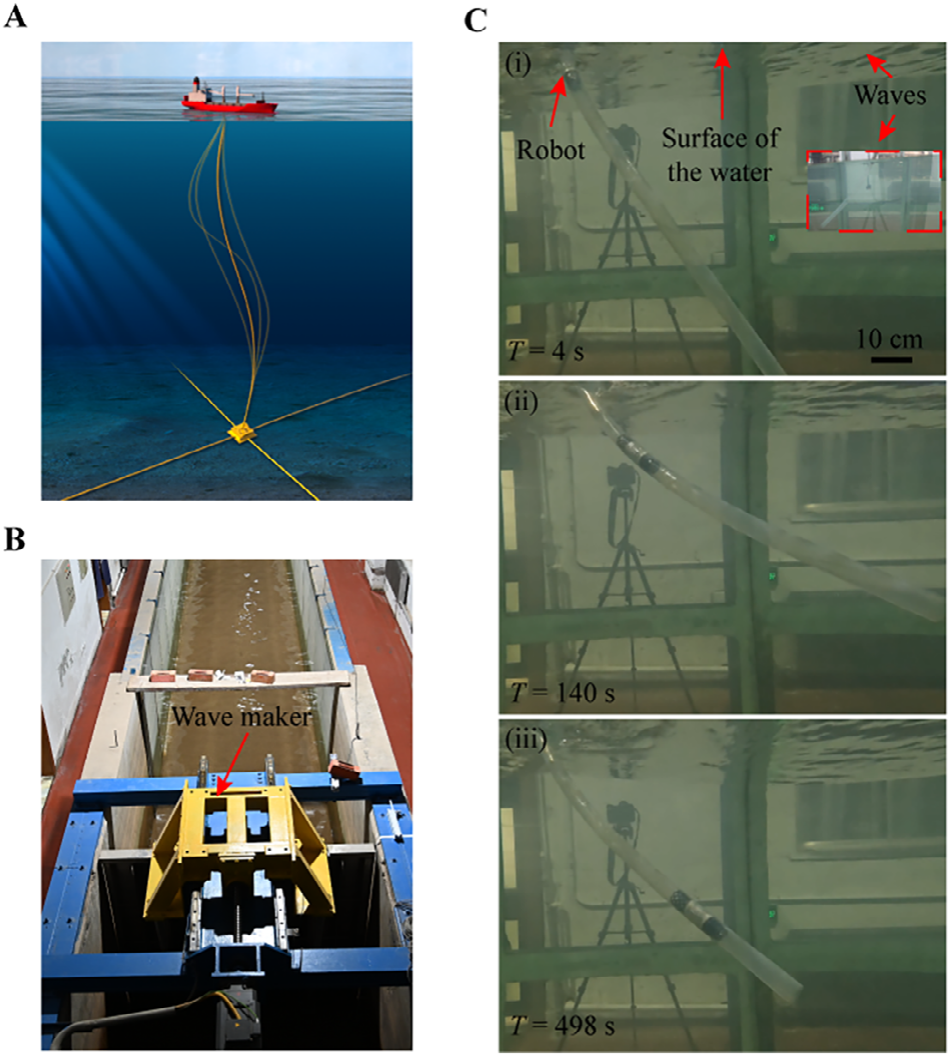
Application of the proposed soft robot in offshore oil and gas development. A) Illustration of offshore oil and gas pipelines. B) Experimental water tank with a wave maker. C) Crawling in an underwater silicone pipe disturbed by waves.

Another potential application of the shell‐lattice crawling robot involves the inspection of offshore oil and gas pipelines, which are critical for the operation of these systems (**Figure** **9**A). These pipelines, extending significant distances underwater, encounter harsh environmental conditions, including constant impacts from ocean waves. Routine inspections are essential to ensure their integrity and operational safety. However, traversing such long and flexible pipelines remains a challenging task for conventional designs. Effective movement requires adaptation to both the fluid dynamics of the marine environment and the pipe deformations caused by external forces.

In a laboratory setting, we simulate the potential application of our designed robot for offshore oil and gas pipelines (Figure 9B). Using a water tank equipped with a wave maker, we generate consistent unidirectional waves with a height of 10 cm and a period of 1.6 seconds. With the pipe left unsealed, the water inside surges in response to the waves. To mimic practical conditions, we introduce additional artificial disturbances to induce oscillations in a silicone pipe, which is fixed at one end above water and allowed to sway freely underwater.

The testing results demonstrate that our soft robot can effectively adapt to the simulated underwater environment and the flexibility of the pipe. The hollow channel reduces the influence of surging fluids during crawling, while its limited bending stiffness allows it to accommodate varying pipe curvatures. As shown in Figure 9C and illustrated in Movie S7 (Supporting Information), the robot maintains robust traversal inside the pipe even amidst random disturbances. This capability underscores its potential to operate reliably in challenging offshore conditions, thereby enhancing maintenance and inspection capabilities that are crucial for the integrity of offshore oil and gas infrastructure.

## 3 Conclusion

In this study, we propose a shell‐lattice soft robot capable of crawling in pipelines with flowing fluids, facilitating inspection tasks without disrupting system operations. The robot features a hollow body design, with a pneumatic actuator in the middle and two lattice shells at the head and tail sections. By periodically applying and releasing negative pressure, the robot achieves an earthworm‐like locomotion through the pipes. This is realized by the design of the head and tail shells with lattice structures to undergo opposite radial deformations, where one is well anchored while the other slides. The thin‐walled hollow design enables the robot to traverse fluid‐filled pipes with high flow velocities. Moreover, the robot can crawl in various types of pipes, accommodating different frictional coefficients, curvatures, and cross‐sectional shapes. Additionally, the robot demonstrates superior load‐bearing capacity, allowing it to operate untethered with a control module and carry functional devices for inspection tasks. These advantages make our designed robot highly promising for the inspection and maintenance of concealed and offshore pipelines.

By incorporating more advanced fabrication techniques, smart materials, and actuation devices, the designed robot may exhibit scalability and applicability across diverse applications. For instance, miniaturized robots could be inserted into soft conduits, such as blood vessels and bile ducts, enabling monitoring and medical interventions without impeding the blood circulation or fluid transportation. The integration of magnetically driven soft materials within the lattice shells, combined with remote magnetic field propulsion, could facilitate free locomotion inside the body. In such scenarios, bidirectional crawling capability might be required for recycling the robot. This could be achieved by incorporating additional lattice shells and actuators, following the proposed shell‐lattice robot design concept. One key challenge lies in designing the loading sequence across the multiple actuators. Another crucial consideration is optimizing the parameters of the lattice shells to generate the necessary frictional forces for initiating crawling. The lattice shells may be subjected to multiple loadings simultaneously, making the determination of parameters more complex. Moreover, optimization of the lattice topology and configuration can enhance crawling capabilities in pipes with varying cross‐sections or significant deformations.

## 4 Experimental Section

### Materials, Modeling, and Fabrication

The basic materials for manufacturing the designed robot are as follows. The hollow actuator consists of two springs made of 65Mn steel (Wenzhou Kaili Spring Hardware Co., Ltd.), two baffles with bulges made of acrylonitrile butadiene styrene (ABS, Stratasys Ltd.), and two waterproof thermoplastic polyurethane (TPU) side fabrics (0.15 mm, Shaoxing Keqiao Gaoyan Knitting and Textile Products Co., Ltd.). The ABS baffles are manufactured using 3D printer Stratasys F170 (Stratasys Ltd.). The lattice shells used in all experiments are made of TPU 92A (Stratasys Ltd.) and are 3D printed using Stratasys F170. To facilitate the assembly process, a ring is integrated at one end of the lattice shells during printing. The connecting plates are made of ABS and are 3D printed using Stratasys F170.

In the preparation stage of 3D printing, the geometric model of the lattice shells and the connecting plates is created in SolidWorks, and then the corresponding STL files are exported for 3D printing. In the proprietary software of the 3D printer, the slicing thickness is set to 0.178 mm, the minimum supporting angle is 45°, and other parameters are set to default values. Additionally, TPU and SR‐30 (Stratasys Ltd.) are used as the model and support materials, respectively. Different nozzles are employed for printing specimens composed of ABS and TPU. During the 3D printing process, TPU is heated to 235 °C in the nozzle and extruded onto the printing platform, enabling rapid solidification of TPU. The material properties of TPU are characterized through uniaxial tension testing. Three standard specimens (ASTM D412‐C) are fabricated, and the resulting stress‐strain curve is shown in Figure S13 (Supporting Information). The Poisson's ratio is obtained from the data provided in Ref. [52].

All the robot specimens are fabricated by assembling the individual components (Figure S6 and Movie S1, Supporting Information). The assembly process is as follows: (i) constructing the framework of the actuator using springs and baffles; (ii) rolling waterproof TPU fabric into two cylinders, attaching them to the inside and outside of the actuator by bonding their ends to the baffles using 502 super glue (Deli Group Co., Ltd.), and sealing both ends of the actuator with silicone sealant (Kafuter K‐705, Guangdong Hengda New Materials Technology Co., Ltd.); and (iii) assembling lattice shells, the actuator, and connecting plates with 502 super glue.

In both the fabrication process and simulations, modeling of the lattice structures proceeds as follows (Figure S3, Supporting Information). First, a quarter part of the unit cell is constructed, where its width, height, and thickness are denoted by *w*, *l*, and *t*, respectively. The angle between the inclined and vertical struts is *α*. The height of vertical struts in re‐entrant and hexagonal unit cells are denoted by *h*_r_ and *h*_h_, respectively. Different *α* are obtained by varying *h*_r_ or *h*_h_, where the re‐entrant and hexagonal unit cells have αr=acrtan(w/w(2hr−l)(2hr−l)) and αr=π−acrtan(w/w(l−2hh)(l−2hh)), respectively. Second, a whole unit cell is obtained by mirroring the quarter part twice, and a planar lattice structure is created by periodically arraying the unit cell along its width and height directions. In the simulation and testing, the number of unit cells in the width direction is set to be 8, and that in the height direction is denoted by *N*. Finally, a lattice shell is formed by rolling the planar lattice structure into a cylinder using its mid‐plane as a reference. Its outer diameter and height are calculated by 16*w*/π+*t* and 2*Nl*, respectively. To prevent buckling of the lattice shells as axial compression is applied, a shell thicknesses larger than 1.5 mm were recommended.

### Numerical Simulations

The numerical simulations are conducted by using Abaqus. In simulations, the lattice shells are composed of TPU, modeled as a linear elastic material with a measured Young's modulus *E* = 30.3 MPa and Poisson's ratio *v* = 0.45.^[^ ^52^ ^]^ To accurately capture the deformation characteristics, the lattice shells are discretized using eight‐node incompatible brick elements (C3D8I), with over three layers of elements along the thickness direction. To predict radial deformations (Figure 2D; Figure S5B, Supporting Information), one end edges of the lattice shells are fixed in the axial direction while allowing free radial expansion and contraction. An axial displacement load is applied to the other end edges to induce stretching and compression of the lattice shells. To evaluate *F*_min_ (Figure 2E; Figure S5C–F, Supporting Information), a glass pipe with a Young's modulus of 70 GPa and a Poisson's ratio of 0.17 is used. In the first step, the lattice shell is inserted into the pipe with an interference fit ranging from 0.21 to 0.70 mm. In the subsequent step, an axial displacement is applied to one end of the lattice shells until sliding occurs, with a frictional coefficient of 0.31 at the contact interface (Figure S9, Supporting Information). The values of *F*_min_ obtained in Figure 2E correspond to the axial reaction forces, while the normal contact forces (Figure S5C–F, Supporting Information) are calculated by multiplying the average contact pressure at all nodes on the contact surface by the contact area. For the detailed parameters of the lattice shells shown in Figure 2E and Figure S5 (Supporting Information), the readers are referred to Table S1 (Supporting Information).

### Measurement of Frictional Coefficients, Frictional Forces, and Contraction Capabilities

In the experiments to measure the frictional coefficients (Figure S9, Supporting Information),^[^ ^12^ ^]^ a box with a layer of TPU adhered to its bottom is dragged to slide on the surface of a plate composed of desired materials. The box is 3D printed and a weight of 100 g is placed on the box. A servomotor equipped with a digital force gauge (AIGU ZP‐5, GTGYGROUP Co., Ltd.) is used to drag the box, at a speed of 0.1 mm s^−1^. When the structure begins to slide, the dragging force is equal to the maximum static frictional force. The total weight of the box determines the normal contact force. The frictional coefficient is then calculated as the ratio between the maximum static frictional force and the normal contact force.

When measuring the frictional forces of the lattice shells in a pipe (Figure 3A,B), a universal testing machine (UTM2203, Shenzhen Suns Technology Stock Co., Ltd.) is employed to apply axial displacement to the end edges of the lattice shells until sliding (Figure S7A,B, Supporting Information). The applied loading rate is 2 mm min^−1^. For each type of lattice shell, three specimens are used for measurement.

In the experiments to measure the contraction capabilities of the actuator (Figure 3C), a syringe is attached to the actuator to adjust the inside pressures. The applied pressures are monitored using a digital pressure gauge (MIK‐Y190, Hangzhou Asmik Sensors Technology Co., Ltd.). To measure the contraction force (Figure S7C, Supporting Information), one end of the actuator is fully fixed while the other end is connected to a fixed force gauge (ELK‐30, ELECALL Electric Co., Ltd.). As pressures are applied, the contraction forces are recorded by the force gauge. To measure the contraction length, one end of the actuator remains fully fixed. A ruler is positioned to measure the remained length of the actuator after contraction. The contraction length is then calculated as the difference between the initial length and the remained length. Three actuators are tested for measurement. In addition, the compressive stiffness of the embedded springs is measured by using the universal testing machine.

### Control Strategy for Crawling

In all experiments, the cyclic loading and unloading of the designed robot are implemented with a vacuum pump and a three‐way valve. The vacuum pump is connected to the chamber of the actuator through an air tube. The vacuum pump applies negative pressure to the actuator, while the three‐way valve is used to release the pressure.

In the untethered design (Figure 7), we integrate a control board (M1, Yiyi Electronics Co., Ltd.), a solenoid valve (JS0520F, Dongwan Jinshunyuan Electronics Co., Ltd.), an air pump (JS370U03PM, Dongwan Jinshunyuan Electronics Co., Ltd.), and a lithium‐ion battery (12v150mAh, Shenzhen Jueyi Technology Co., Ltd.) inside the robot and secure them at one end of the actuator. All electronic components are powered by the lithium‐ion battery. The connections between these devices are illustrated in Figure S14 (Supporting Information). The control board includes a microcontroller and a relay. The operator can wirelessly interact with the microcontroller to remotely control the states of the relay, which controls the operation of the air pump and solenoid slave with three interfaces. The relay has two working states. In one state, the air pump works while the solenoid valve is inactive. In the other state, the solenoid valve is activated while the air pump is inactive. These states correspond respectively to applying and releasing air pressure within the actuator, enabling contraction and extension. To suppress the electromagnetic interference generated by the solenoid valve, a diode is incorporated into the circuit.

### Notes on Experimental Setup

The U‐shaped rigid pipes used to evaluate the minimum turning radius consist of two straight segments and one curved segment (Figure S10, Supporting Information). Each segment has a central axis length of 200 mm. The curved segments feature constant curvatures, with the radii of 80 mm, 100 mm, 120 mm, 140 mm, and 160 mm. The segments are made of polylactic acid (PLA, Bambu Lab.). With a stiffness of 2.9 GPa, which is ≈100 times greater than the robot body, the rigid pipes undergo negligible deformation. Each segment is individually 3D printed using the 3D printer from Bambu Lab X1‐Carbon, and then all the segments are assembled by gluing.

The oil used in Figure 4E is 100# vacuum pump oil (Dongguan Yingfeite Lubrication Technology Co., Ltd.). The UTL solution is from BMF Material Technology Inc. The uncured silicone rubber is a mixture of Ecoflex 0030 (Smooth‐On Inc.), prepared by thoroughly mixing two liquid components in a 1:1 weight ratio.

In the experiment to test the crawling capability in the double‐arc curved pipe (Figure 4F), a variable‐frequency pump (SA‐2000, Sobo) is used to regulate the flow rate inside the pipe. The pipe consists of three curved sections, each with a curvature radius of 14 cm, and one straight section. Each segment has a length of 20 cm. These segments are glued together to form a serpentine pipeline. The fluid flow rate is measured using a turbine flow meter (LLJ‐25, Yongjia Aocheng Hardware Products Co., Ltd.).

## Conflict of Interest

The authors declare no conflict of interest.

## Author Contributions

D.G.: conceptualization, investigation, methodology, experimental work, formal analysis, visualization, and writing–original draft. Y.W.: conceptualization, investigation, methodology, research supervision, and writing–review and editing. Z.K.: conceptualization, investigation, research supervision, and writing–review and editing.

## Supporting information



Supporting Information

Supplemental Movie 1

Supplemental Movie 2

Supplemental Movie 3

Supplemental Movie 4

Supplemental Movie 5

Supplemental Movie 6

Supplemental Movie 7

## Data Availability

The data that support the findings of this study are available in the supplementary material of this article.
