# Supplementary material for: A Hollow Shell‐Lattice Soft Robot in Flexible Pipelines with Flowing Fluids
Source: Adv Sci (Weinh). 2025 Mar 27;12(21):2414882. doi: 10.1002/advs.202414882 (PMC12140295; doi:10.1002/advs.202414882)
Supplement: Supplementary file 1 — Supporting Information [file ADVS-12-2414882-s004.pdf]

ADVANCED SCIENCE
Open Access

Supporting Information

for *Adv. Sci.*, DOI 10.1002/advs.202414882

A Hollow Shell-Lattice Soft Robot in Flexible Pipelines with Flowing Fluids

Di Guo, Yiqiang Wang and Zhan Kang**

Supporting Information for

A Hollow Shell-Lattice Soft Robot in Flexible Pipelines with Flowing Fluids

Di Guo[1], Yiqiang Wang[1]*, Zhan Kang[1]*

[1]State Key Laboratory of Structural Analysis, Optimization and CAE Software for Industrial Equipment, Dalian University of Technology, Dalian 116024, China

*Corresponding authors. Yiqiang Wang Email: wangyq@dlut.edu.cn
Zhan Kang Email: zhankang@dlut.edu.cn

Supplementary Notes

Note S1. Drag force of hollow cylinders within a fluid pipe

The drag forces acting on the hollow cylindrical soft robot in a fluid-flowing pipe are calculated using ANSYS Fluent (Figure S1A). In the finite-volume model, the fluid pipe has a diameter of 32 mm and a length of 1500 mm, and is discretized by 4-node tetrahedral elements. The robot body is modeled as a hollow solid-material cylinder. Its height and outer diameter are respectively 71 mm and 32 mm, and its inner diameter varies from 8 mm to 24 mm. The fluid density is 1000 kg/m^3, and the viscosity is 1.01×10^{-3} kg/(m·s). The average flow velocity is specified at the inlet of the pipe, and the outlet pressure is prescribed as 0 Pa. The robot is positioned at a distance of 1000 mm from the inlet of the pipe, which ensures that a fully-developed flow is formed. As the Reynolds numbers exceed 15000, the fluid inside the pipe is turbulent. Therefore, the standard k-ε turbulence model is employed to simulate the fluid flow.[1] The hollow cylinders are modeled as rigid bodies.

The numerical simulation results show that the drag force increases with decreasing inner diameter and increasing fluid velocity (Figure S1B). At an inlet velocity of 0.5 m/s, the drag forces are 40.24 N and 0.15 N for the cylinders with inner diameters of 8 mm and 24 mm, respectively. As the inlet velocity increases to 1.5 m/s, the corresponding drag forces increase to 351.24 N and 1.35 N, respectively.

The drag forces of the robot crawling in different fluids have also been numerically analyzed. In these simulations, the lattice model is used instead of the cylindrical shells, with all other parameters and setting remaining consistent with those previously described. Figures S1C and S1D present the drag forces and the frictional resistance of the flowing fluids acting on the robot, respectively. The former represents the total force exerted on all contact surfaces between the robot and fluid, while the latter accounts for the forces acting on the inner surfaces of the lattice shell and those of the hollow actuator. For crawling in the water-filled pipe, the drag force increases from 0.07 N to 16.46 N, and the fluid frictional force increases from 0.002 N to 0.25 N, as the inlet velocity increases from 0.1 m/s to 1.59 m/s. For crawling in the pipe filled with uncured silicone

rubber (Ecoflex 0030), the drag force increases from 10.61 N to 208.28 N, and the frictional force increases from 2.62 N to 47.31 N, as the inlet velocity increases from 0.1 m/s to 1.59 m/s.

Note S2. Mechanical analysis for minimum axial forces to slide cylindrical shells

The minimal axial force to slide a single cylindrical shell against the frictional force acted by the pipe wall is estimated. The cylindrical shells are characterized by their outer diameter D, wall thickness t, and height h (Figure S2A). They are composed of isotropic basic materials with Young's modulus E and Poisson's ratio v. An interference fit is prescribed between the cylindrical shell and the pipe wall. Provided the inner radius of the pipe R, the interference amount is obtained as $\delta D = D - 2R$. The frictional coefficient of the interface between the pipe and the cylindrical shell is denoted by μ. In the following calculations, the stiffness of the pipe is assumed to be much higher than that of the cylindrical shell, allowing us to neglect the deformation of the pipe. The following derivation assumes that the shell uniformly deforms in the radial directions with respect to the applied axial force.

Two forces are applied to the cylindrical shell in the pipe. One is the radial pressure q acted by the pipe (Figure S2B). It is caused by the interference fit. Here, the hoop strain and hoop stress of the cylindrical shell are determined by $\varepsilon_\theta = -\delta D / \left(D - t\right)$ and $\sigma_\theta = E\varepsilon_\theta$, respectively. Considering the equilibrium condition for a cylindrical shell subjected to the contact pressure,[2] one has $q = -2t\sigma_\theta / D$. By multiplying the contact area $A_c = \pi Dh$, the normal contact force is obtained by

$$F_N^0 = 2Et\delta DA_c / \left(D^2 - Dt\right) \tag{S1}$$

where the superscript '0' indicates the state that only radial contact pressure is applied to the cylindrical shell.

Besides the radial contact pressure, an axial force F is applied on the edges of the cylindrical shells. This force will cause either radial expansion or radial contraction of the shells, with the hoop strain expressed by

$$\varepsilon_F = -vF / \left(A_s E\right) \tag{S2}$$

where $A_s = \pi(D-t)t$ is the cross-sectional area of the cylindrical shell.

The radial expansion and contraction also cause a radial contact pressure change, denoted by $\tilde{q}$. Specifically, it introduces a hoop strain of

$$\varepsilon_{\tilde{q}} = -\tilde{q}D/(2Et) \tag{S3}$$

Because the cylindrical shells maintain tight contact with the pipe wall during axial loading, the total hoop strain caused by the axial force F and the contact pressure change $\tilde{q}$ satisfies $\varepsilon_F + \varepsilon_{\tilde{q}} = 0$ (Figure S2C). Accordingly, by combining Eqs. (S2) and (S3), the contact pressure change is obtained as $\tilde{q} = -2Fvt/(A_s D)$. Subsequently, the normal contact force under the applied axial force becomes

$$F_N = \frac{2Et\delta D}{D^2 - Dt} A_c - \frac{2Fvt}{A_s D} A_c \tag{S4}$$

The cylindrical shells begin to slide on the condition that the applied axial force exceeds the frictional force:

$$|F| > \mu F_N \tag{S5}$$

where $|F|$ indicates the absolute magnitude of the applied force.

As a tensile loading is applied, the minimum axial force to slide the cylindrical shell is

$$F_{min}^{t} = (2\pi\mu Eth\delta D)/(D - t + 2\mu vh) \tag{S6}$$

while for an applied compressive loading, it becomes

$$F_{min}^{c} = -(2\pi\mu Eth\delta D)/(D - t - 2\mu vh) \tag{S7}$$

where Eq. (S6) and (S7) hold under the condition that $D - t > |2v\mu h|$, otherwise, the increase in the frictional force will exceed the applied force.

It is observed that F_{min} is dependent on the Poisson's ratio v of the basic material. In the case v = 0, we have $|F_{min}| = 2\pi\mu Eth\delta D/(D-t)$ for both axial tension and compression. In the cases with non-zero v, the corresponding minimal axial forces are rewritten as

$$F_{min} = \begin{cases} \dfrac{\psi}{1+kv} & \text{for tensions} \\ -\dfrac{\psi}{1-kv} & \text{for compressions} \end{cases} \tag{S8}$$

with $\psi = 2\pi\mu Eth\delta D/\left(D-t\right)$ and $k = 2\mu h/\left(D-t\right)$.

Note S3. Measurement of flow rate and calculation of flow speed

In the experiment to test the crawling capability in fluid pipes (Figure 4 and Movie S2), we employ a pump (ACP-15000, Zhangzhou Healthy Aquarium Products Co., Ltd) to supply water to the glass pipe. The volume flow rate Q is measured by a turbine flow meter (LLJ-25, Yongjia Aocheng Hardware Products Co., Ltd.). The average flow speed is calculated by $V = Q/(\pi R^2)$.

Note S4. Mechanical analysis of maximal payload as crawling in a vertical pipeline

The load-bearing capability of the robot within a vertical pipe is determined by the difference of the frictional forces between the head and tail lattice shell sections. Without the payload, the minimal axial forces to slide the head lattice under tensile and compressive forces are denoted by $\left(F_{\min}^{\mathrm{t}}\right)_{\mathrm{Head}}$ and $\left(F_{\min}^{\mathrm{c}}\right)_{\mathrm{Head}}$, and those for the tail lattice are $\left(F_{\min}^{\mathrm{t}}\right)_{\mathrm{Tail}}$ and $\left(F_{\min}^{\mathrm{c}}\right)_{\mathrm{Tail}}$, respectively. The payload is placed on the connecting plate at the end of the head lattice (Figure S12A), and can be regarded as a tensile force G applied to the head section (Figure S12B). Due to the relatively small self-weight of the lattice shell sections, their influence on the minimal sliding force is considered negligible.

Depending on the magnitude of G, the following two scenarios are considered: (1) For the case of $G > \left(F_{\min}^{\mathrm{t}}\right)_{\mathrm{Head}}$, the head lattice section cannot be anchored to the pipe wall. Therefore, the robot cannot carry the payload while crawling upward in the vertical pipe. (2) For the case of $G < \left(F_{\min}^{\mathrm{t}}\right)_{\mathrm{Head}}$, the head lattice shell section can be tightly anchored, where the minimal axial tensile and compressive forces to slide the head lattice become $\left(F_{\min}^{\mathrm{t}}\right)_{\mathrm{Head}} - G$ and $\left(F_{\min}^{\mathrm{c}}\right)_{\mathrm{Head}} + G$, respectively. In this case, three locomotion modes can be triggered depending on the magnitude of G.

In the first mode, we have $G < \min\left\{\left(F_{\min}^{t}\right)_{\text{Head}} - \left(F_{\min}^{t}\right)_{\text{Tail}},\ \left(F_{\min}^{c}\right)_{\text{Tail}} - \left(F_{\min}^{c}\right)_{\text{Head}}\right\}$. Under the applied tensile force, it yields $\left(F_{\min}^{t}\right)_{\text{Head}} - G > \left(F_{\min}^{t}\right)_{\text{Tail}}$. This indicates that the tail section slides upward while the head section remains anchored to the pipe wall. In the compressive loading condition, it holds $\left(F_{\min}^{c}\right)_{\text{Head}} + G < \left(F_{\min}^{c}\right)_{\text{Tail}}$. This indicates that the tail section becomes anchored while the head section is pushed upward. Through a cyclic loading/unloading process, the robot crawls in the upward direction.

The second mode occurs when $\min\left\{\left(F_{\min}^{t}\right)_{\text{Head}} - \left(F_{\min}^{t}\right)_{\text{Tail}},\ \left(F_{\min}^{c}\right)_{\text{Tail}} - \left(F_{\min}^{c}\right)_{\text{Head}}\right\} < G < \max\left\{\left(F_{\min}^{t}\right)_{\text{Head}} - \left(F_{\min}^{t}\right)_{\text{Tail}},\ \left(F_{\min}^{c}\right)_{\text{Tail}} - \left(F_{\min}^{c}\right)_{\text{Head}}\right\}$. In this case, either the head or the tail lattice shell section cannot be anchored to the pipe wall under both tensile and compressive loading conditions. As a result, only one section is dragged and pushed during the cyclic loading process. The robot is unable to crawl in the pipe.

In the third locomotion mode, the payload exceeds the difference in $F_{\min}$ between the two lattice shell sections, i.e. $G > \max\left\{\left(F_{\min}^{t}\right)_{\text{Head}} - \left(F_{\min}^{t}\right)_{\text{Tail}},\ \left(F_{\min}^{c}\right)_{\text{Tail}} - \left(F_{\min}^{c}\right)_{\text{Head}}\right\}$. In the tensile loading condition, it has $\left(F_{\min}^{t}\right)_{\text{Head}} - G < \left(F_{\min}^{t}\right)_{\text{Tail}}$, which implies that the head section begins to slide while the tail section is anchored. In the compressive loading condition, we have $\left(F_{\min}^{c}\right)_{\text{Head}} + G > \left(F_{\min}^{c}\right)_{\text{Tail}}$, indicating that the head section is anchored but the tail section slides. As a result, the robot crawls in the downward direction.

In summary, the maximum load-bearing capability of the robot crawling in a vertical pipe is $G = \min\left\{\left(F_{\min}^{t}\right)_{\text{Head}} - \left(F_{\min}^{t}\right)_{\text{Tail}},\ \left(F_{\min}^{c}\right)_{\text{Tail}} - \left(F_{\min}^{c}\right)_{\text{Head}}\right\}$.

Note S5. Method to estimate the endurance of the robot in untethered mode

In the untethered design, a battery is required to power a solenoid valve, an air pump, and the control board. The relay in the circuit has two states, corresponding to the operation of either the air pump or the solenoid valve. In other words, the battery supplies power either to the combination of the valve and control board or to the combination of the pump and control board.

Assuming identical power consumption for all components, let P_1 and P_2 represent the power consumption of the valve (or pump) and that of the control board, respectively. The total power consumption of the robot can then be expressed as $P = P_1+P_2$. Given a battery capacity of W, the estimated endurance time is given by $t_{max} = W/P$. The maximum crawling distance can be estimated as $d = t_{max}V_r$, where V_r represents the crawling speed of the robot.

For instance, in the untethered design in Figure 7, the battery is required to power a solenoid value (12V, 2W), an air pump (12V, 2W), and a control board (12V, 0.5W). If a 12V, 300 mAh battery is equipped, the robot can crawl continuously for approximately 86.4 minutes. Based on the tested crawling speed of 1.96 mm/s, the robot can travel a distance of 10.16 m.

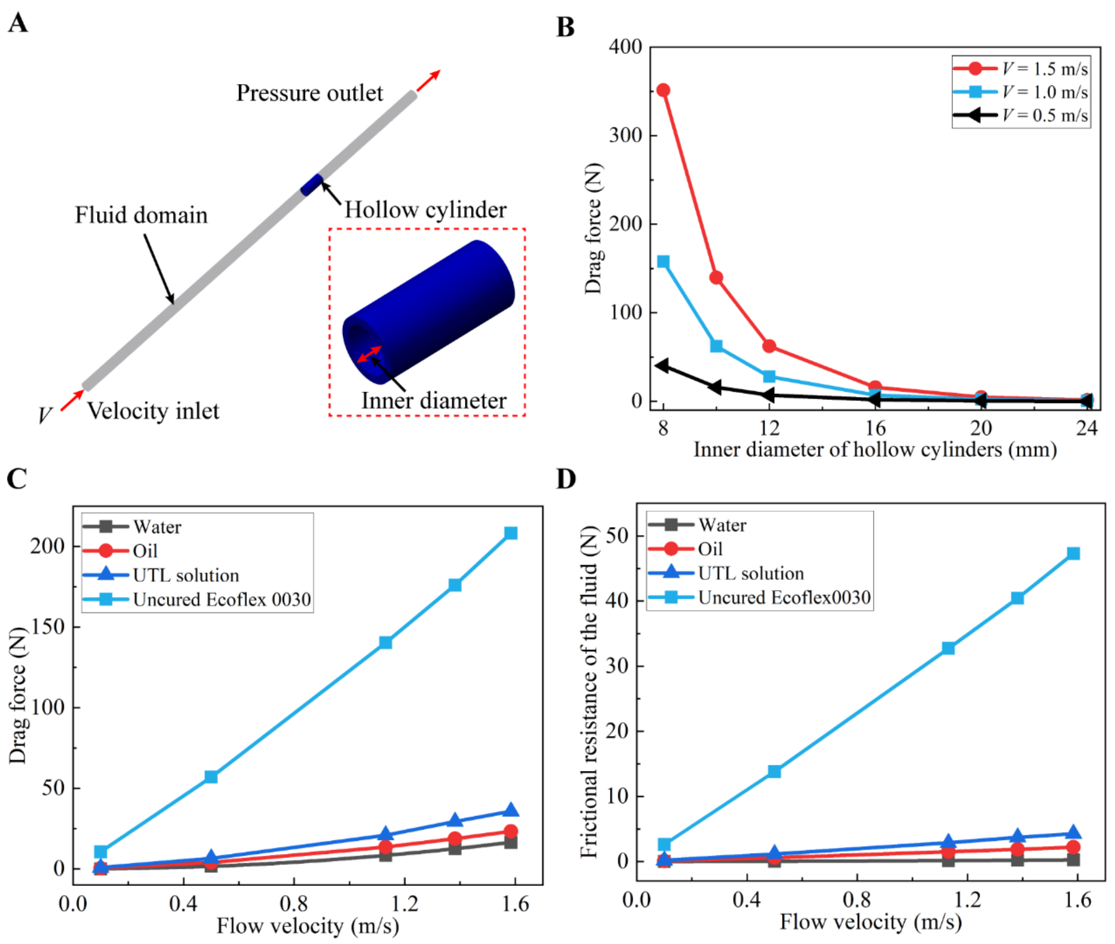

Figure S1. Drag force for hollow cylinders and shell-lattice robots within fluid pipes. (A) Simulation model. The gray regions indicate the fluid domain, and the blue cylinder indicates the robot body. (B) Variation of the drag forces versus the inner diameters of the hollow cylinders where the inner diameter of the pipe and the outer diameter of the hollow cylinders are 32 mm. (C) Variation of drag forces of the hollow shell-lattice robot versus the velocities of flowing fluids with different viscosities. (D) Variation of frictional resistance of fluids on the hollow shell-lattice robot versus the velocities of flowing fluids with different viscosities.

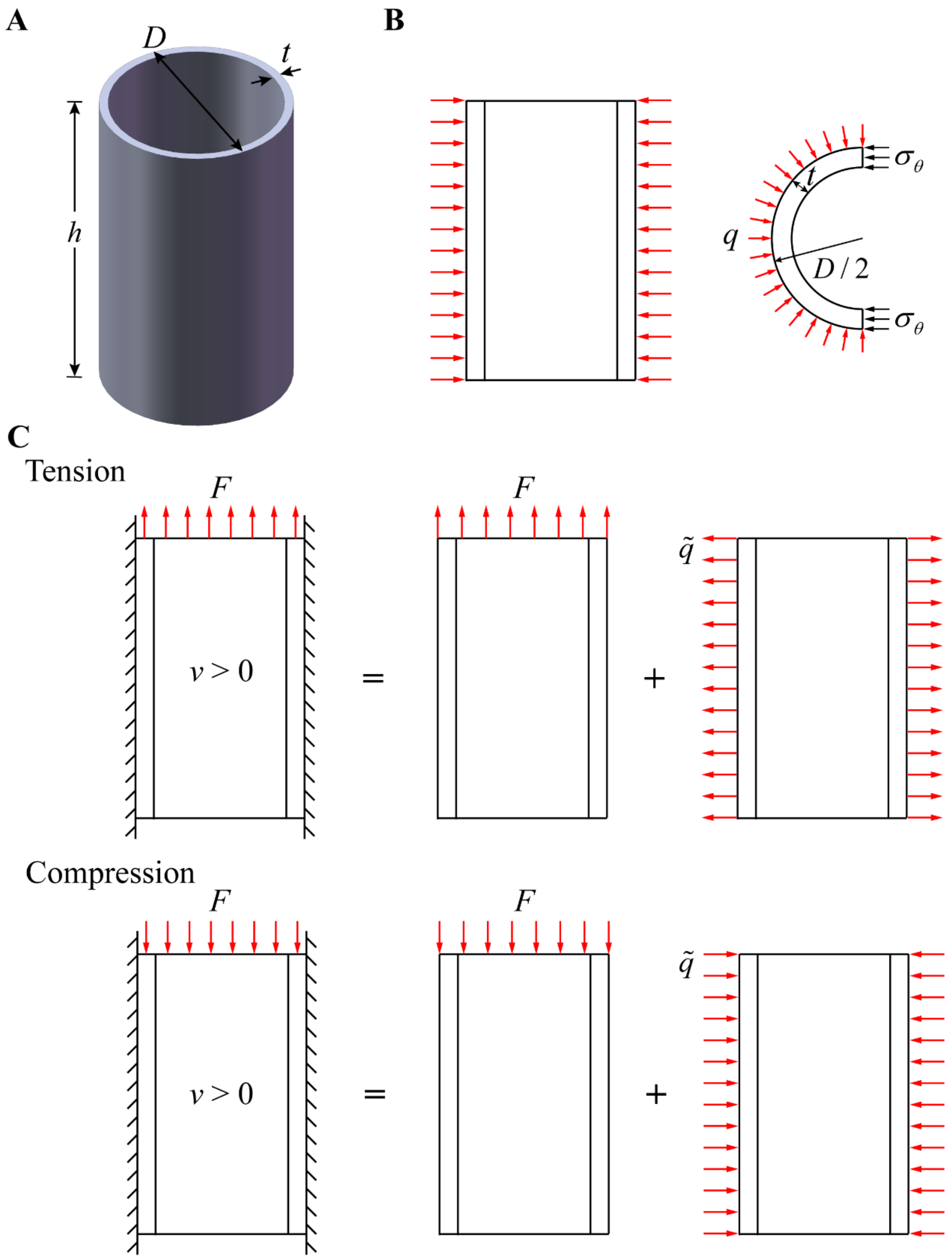

Figure S2. Mechanics model of a cylindrical shell in a pipe under different loading conditions. (A) Geometric model of the cylindrical shells. (B) Mechanics model of the cylindrical shells under interference fit conditions, where the outer surface is subjected to contact pressure. (C) Mechanics models of the cylindrical shells in a pipe subjected to axial forces at one end, where their mechanical behaviors are considered as a linear superposition of two separate loading cases.

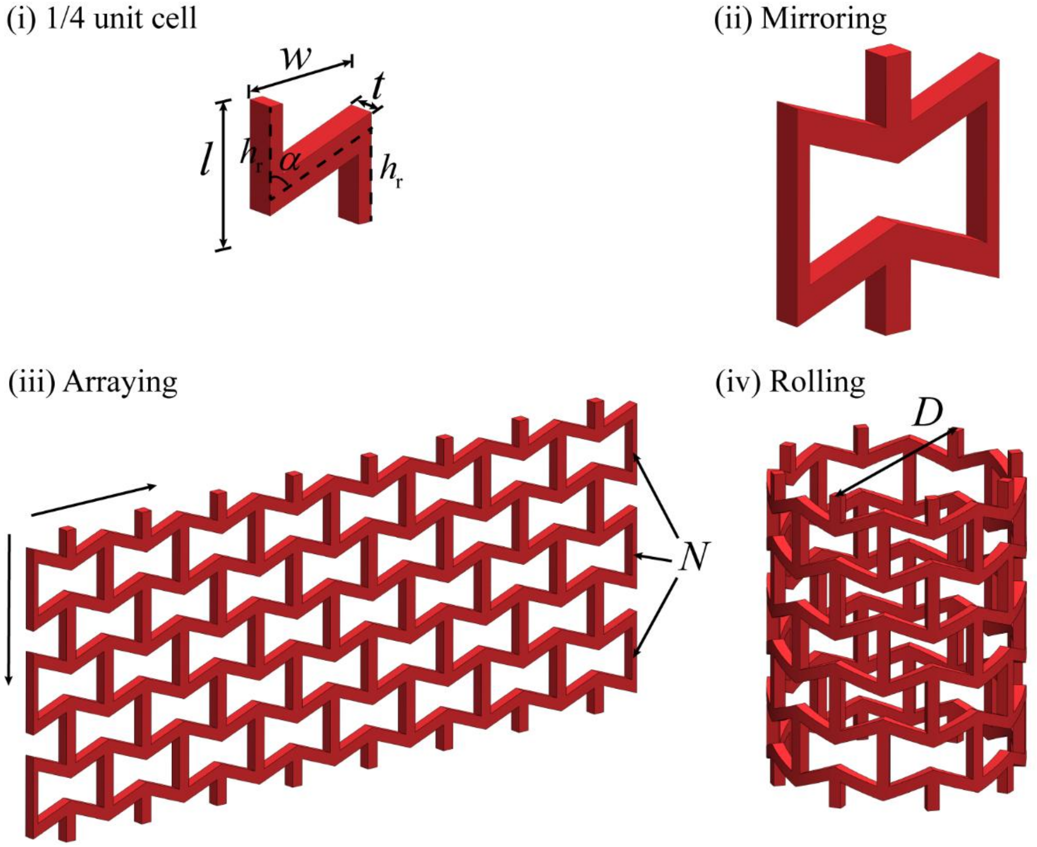

Figure S3. Geometric modeling of a lattice shell section

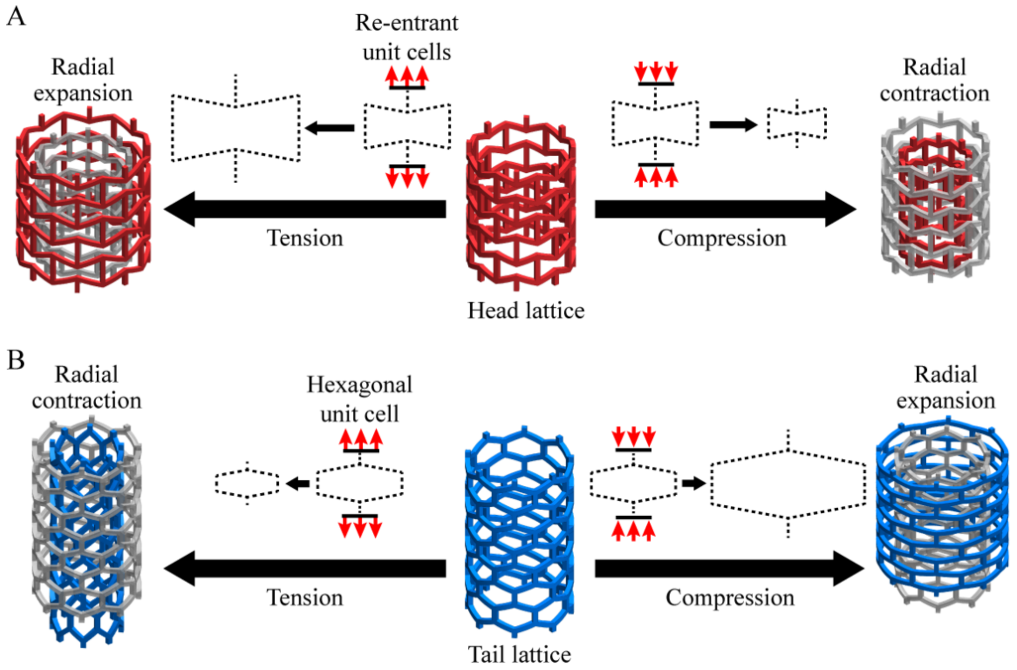

Figure S4. Deformation mechanisms of the (A) re-entrant and (B) hexagonal lattice shells under uniaxial compression and tension, which are governed by the deformations of their unit cell architectures.

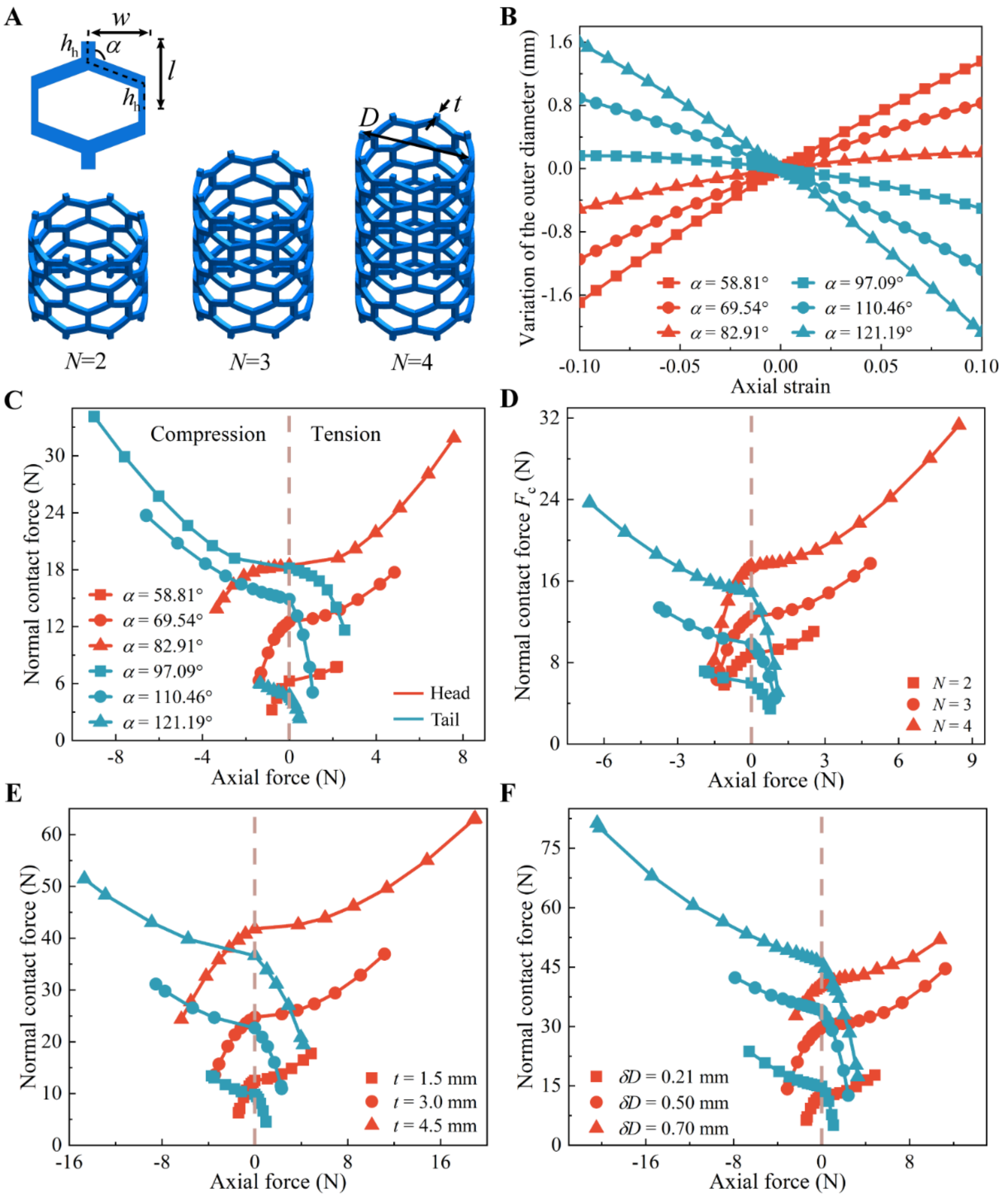

Figure S5. Mechanical behaviors of lattice shells with different parameters under axial forces. (A) Geometric model of lattice shells. (B) Simulated variation of the outer diameter with axial strain for lattice shells with different angles α, where other geometric parameters remain unchanged. Variation of the normal contact forces with the applied axial forces for lattice shells with different (C) angles α, (D) numbers of unit cells along the axis N, (E) wall thicknesses t, and (F) interference amounts δD.

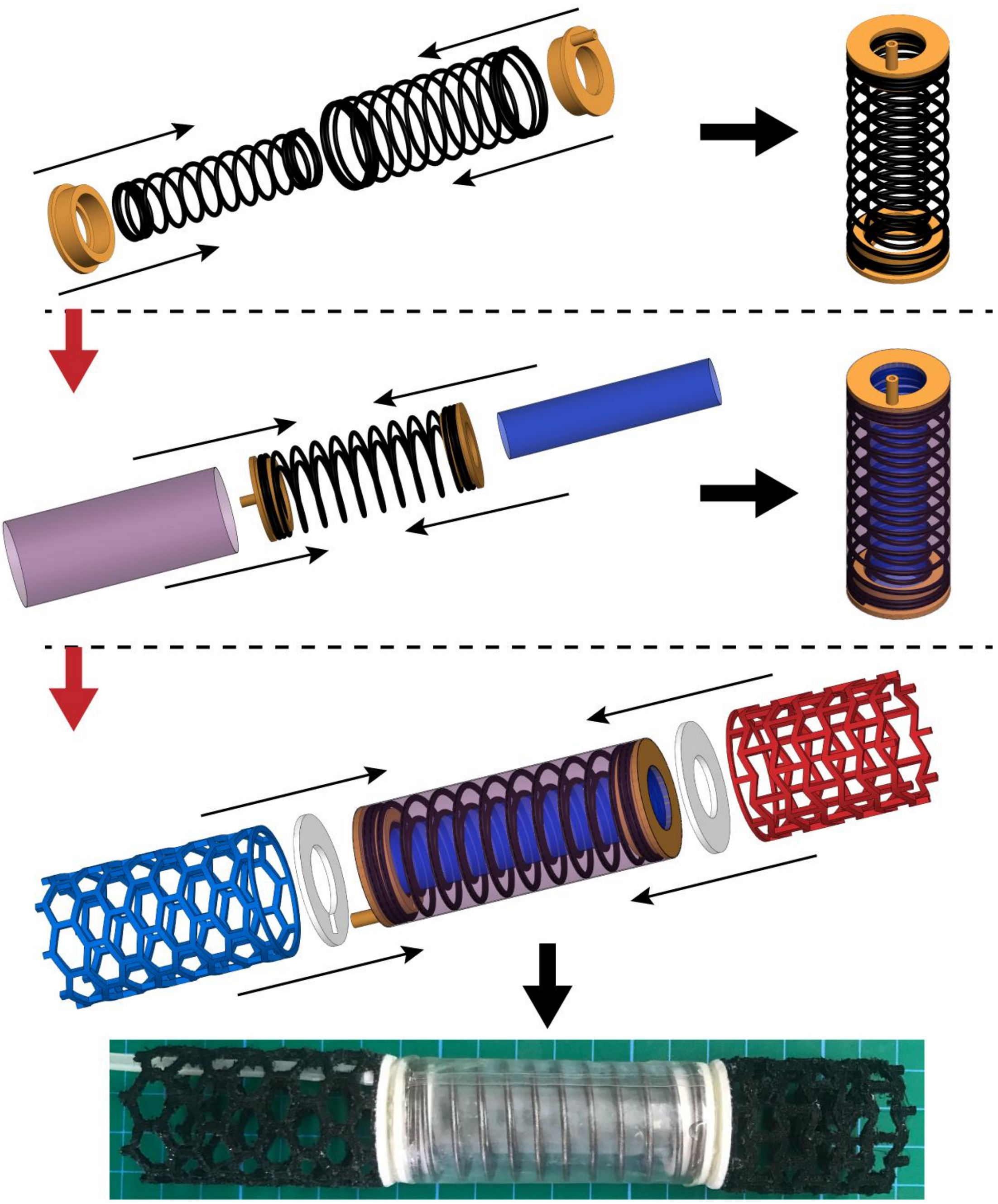

Figure S6. Assembly process of shell-lattice soft robots.

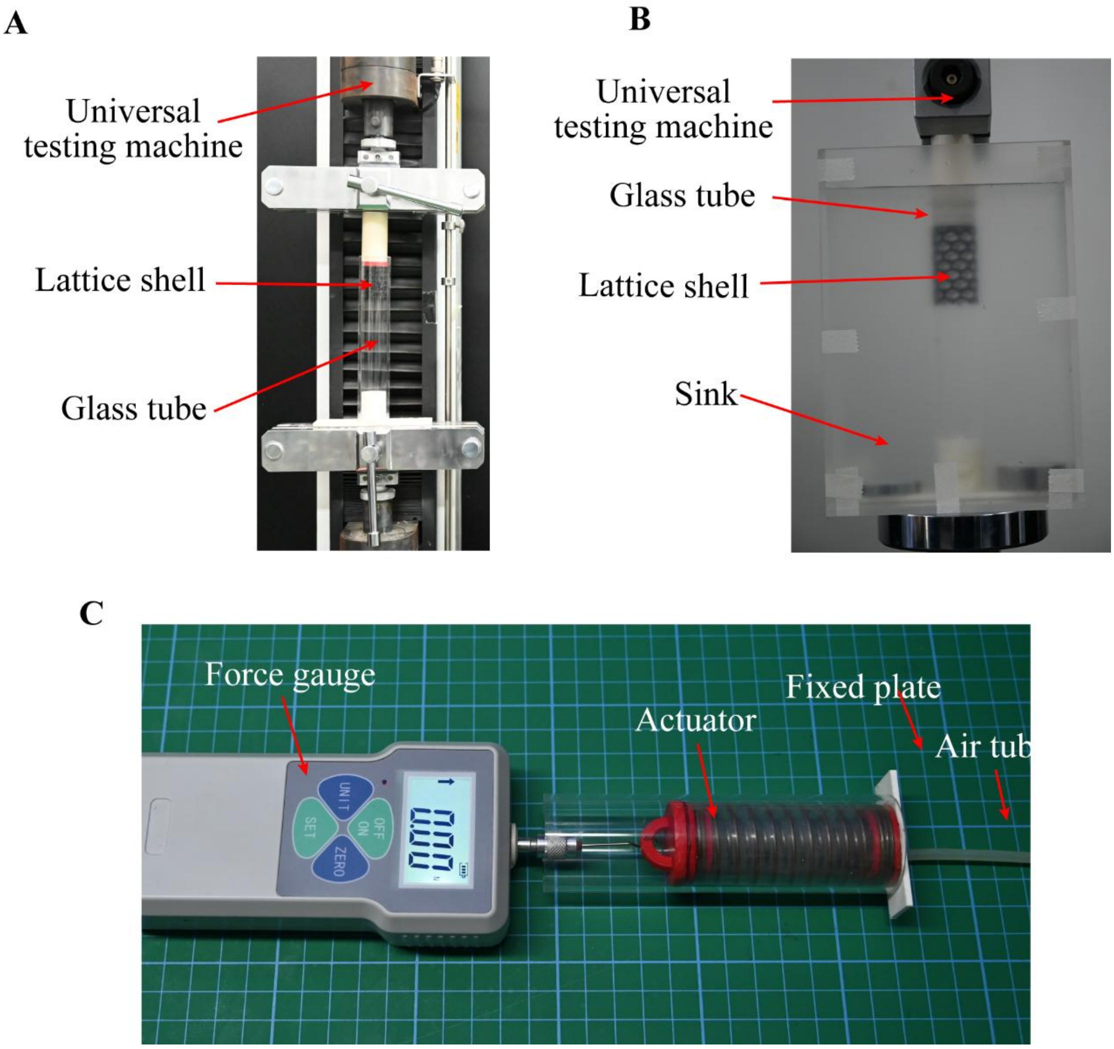

Figure S7. Experimental setup for measurement of (A) frictional forces of lattice shells within a glass tube; (B) within a glass tube submerged underwater; and (C) the contraction capability of the pneumatic actuator.

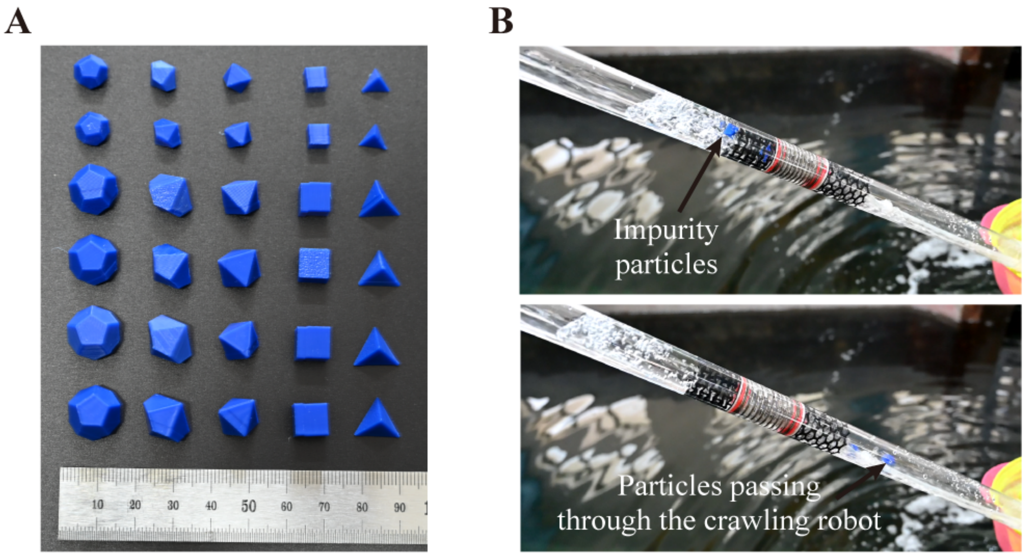

Figure S8. Crawling in a pipe with flowing fluid mixed with impurity particles (A) 3D-printed particles with varying sizes. (B) Particles passing through the crawling robot.

A

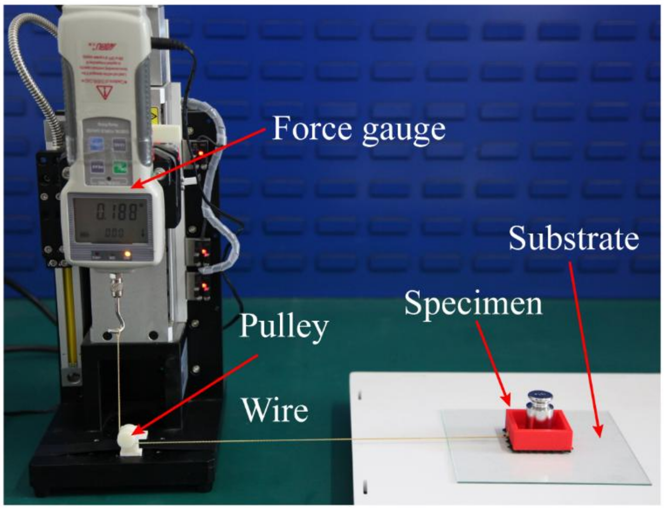

B

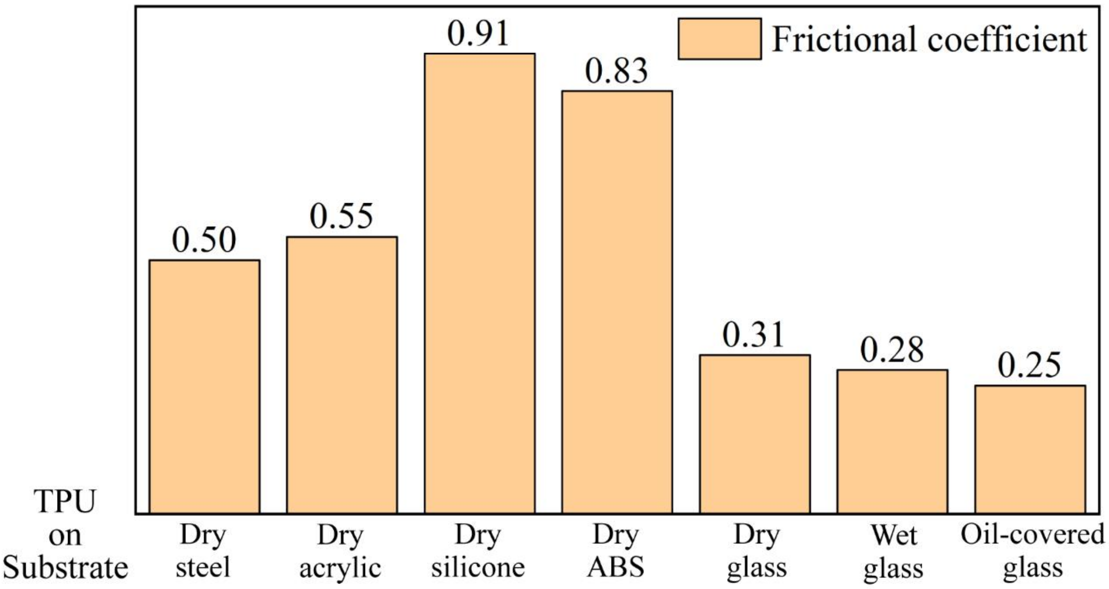

Figure S9. Measurement of frictional coefficients. (A) Experiment setup for frictional coefficient measurement. (B) Measured frictional coefficients between TPU and various substrates.

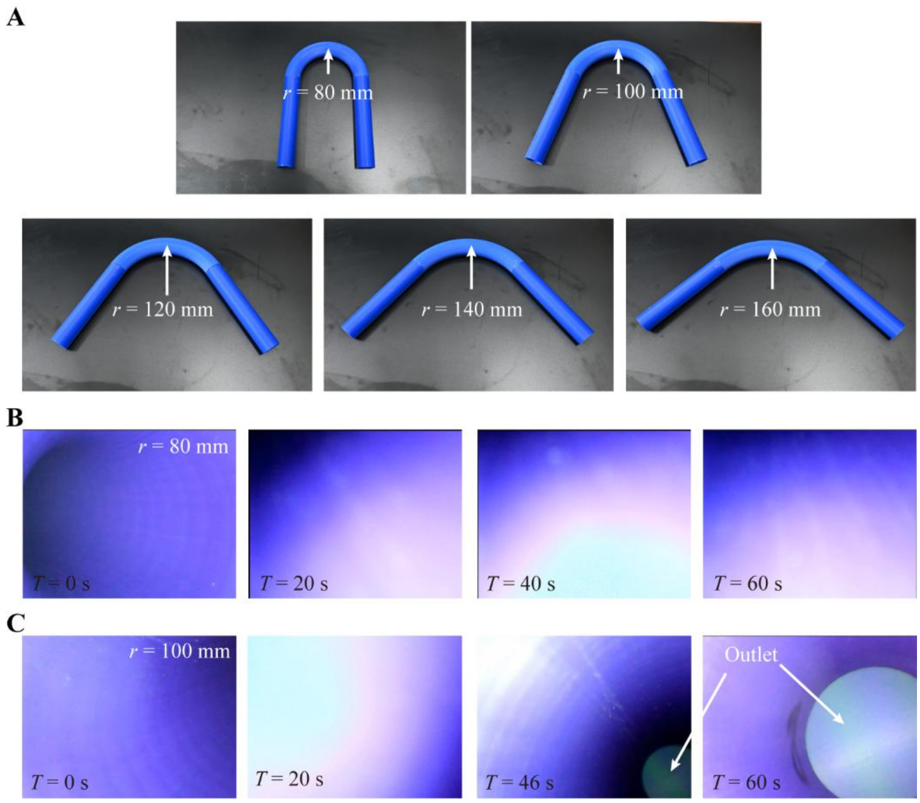

Figure S10. Experiment on crawling in rigid curved pipes with different curvature radii. (A) Rigid curved pipes with marked curvature radii. Endoscopic images captured during the crawling process in pipes with radii of (B) 80 mm and (C) 100 mm. In (B), the field of view from the endoscope does not capture the outlet of the U-shaped pipe, indicating that the robot fails to crawl through. In (C), the endoscope successfully captures the outlet, suggesting that the robot is able to crawl through the pipe.

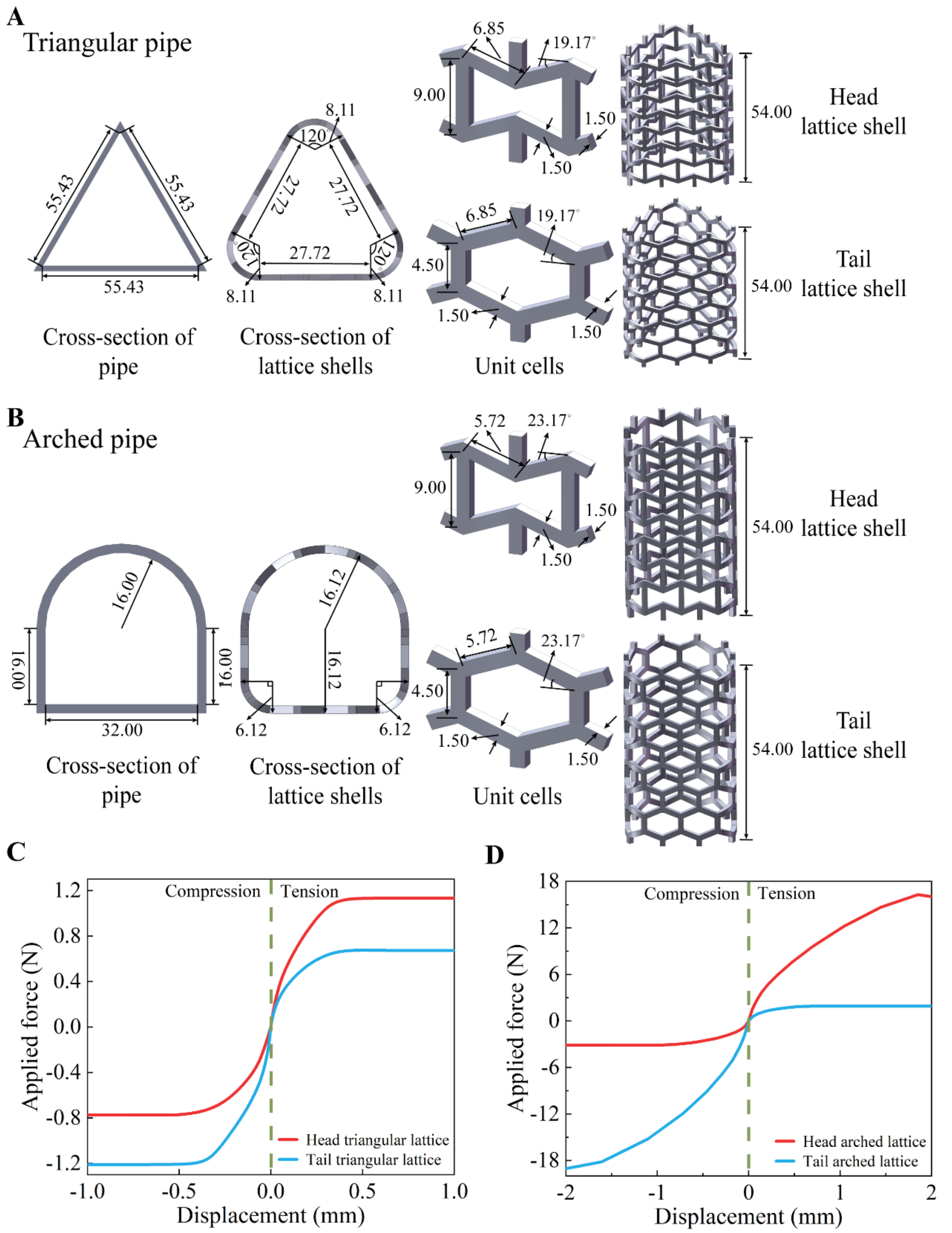

Figure S11. Structural design and mechanical properties of lattice shells with triangular and arched cross-sections. Geometric models of lattice shells with (A) a triangular cross-section and (B) an arched cross-section for pipes with corresponding cross-sectional shapes. Simulated force-displacement curves for lattice shells within (C) triangular pipes and (D) arched pipes under axial loads.

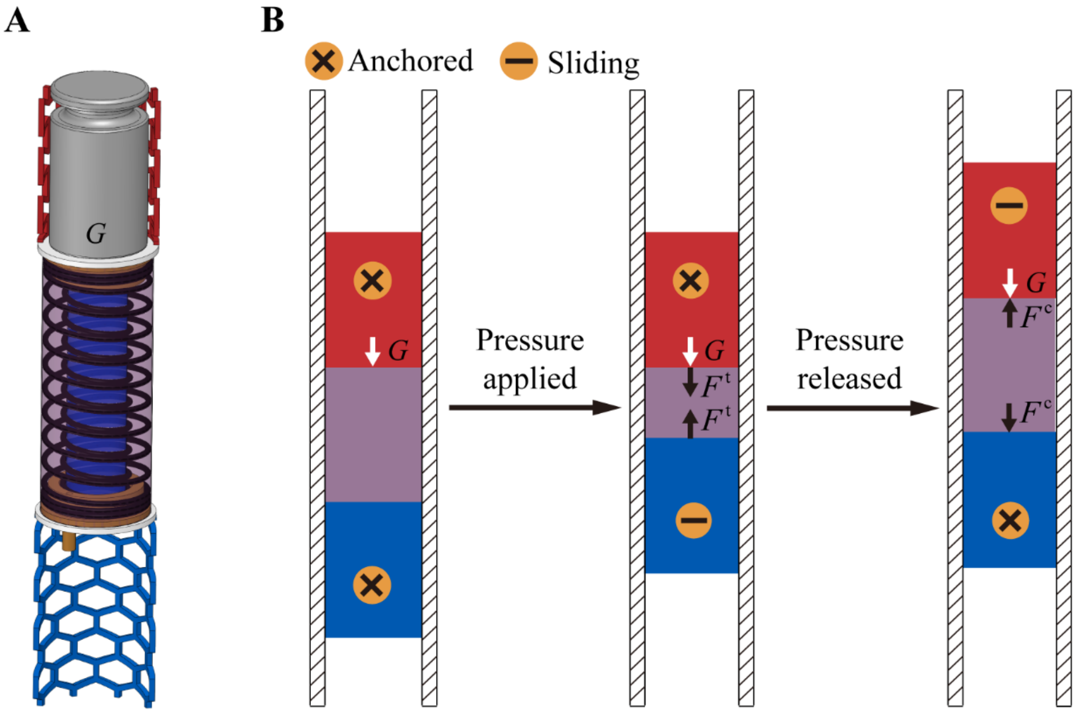

Figure S12. Mechanical model of shell-lattice soft robots crawling with a payload. (A) Schematic illustration of a soft robot with a payload. (B) Mechanical analysis of the soft robot crawling with a payload in a vertical pipe.

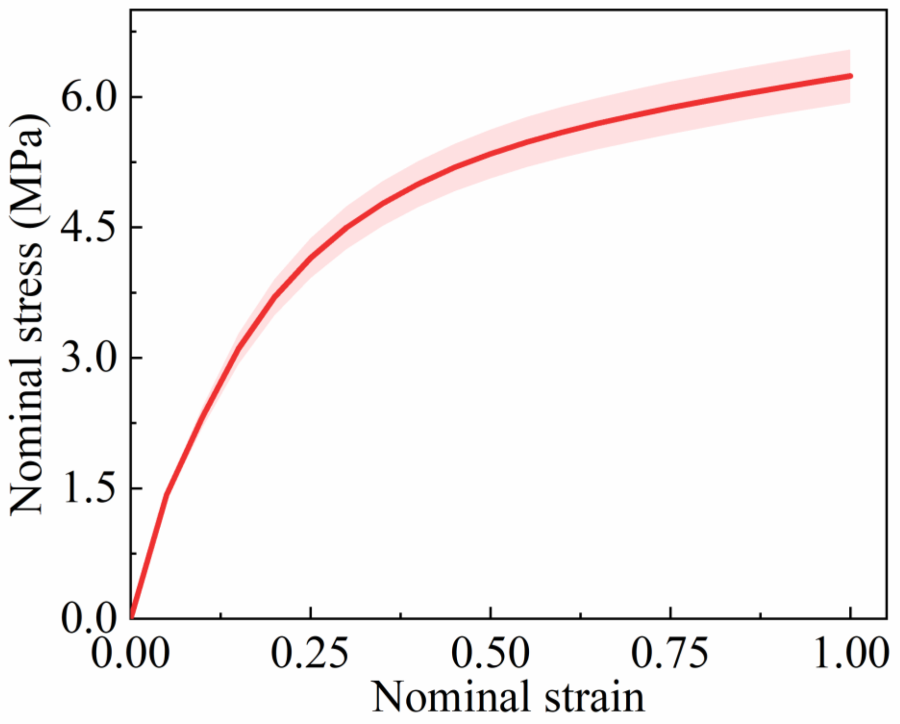

Figure S13. Stress-strain curve for 3D-printed TPU under uniaxial tension. The solid line is the average value, and the shadow regions indicate the standard deviation from three specimens.

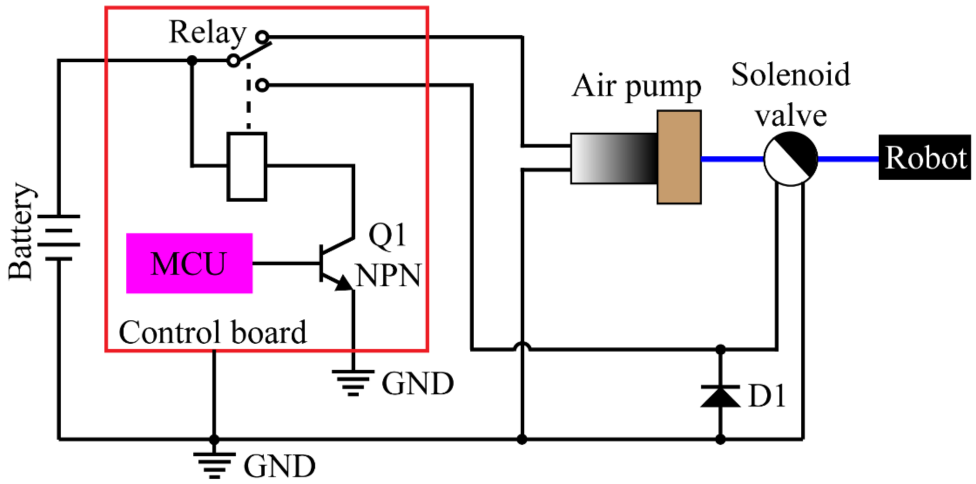

Figure S14. Control system of untethered shell-lattice soft robot.

Table S1. Parameters of the lattice shells in Figure 2E and Figure S5

<table>
<tr><th></th><th>α [°]</th><th>N</th><th>t [mm]</th><th>δD [mm]</th><th>Young's modulus E [MPa] 1)</th><th>Cell size factor λ 2)</th></tr>
<tr><td rowspan="3">Head lattice</td><td>58.81</td><td rowspan="6">3</td><td rowspan="6">1.5</td><td rowspan="6">0.21</td><td rowspan="24">30.30</td><td rowspan="30">1</td></tr>
<tr><td>69.54</td></tr>
<tr><td>82.91</td></tr>
<tr><td rowspan="3">Tail lattice</td><td>97.09</td></tr>
<tr><td>110.46</td></tr>
<tr><td>121.19</td></tr>
<tr><td rowspan="3">Head lattice</td><td rowspan="3">69.54</td><td>2</td><td rowspan="6">1.5</td><td rowspan="6">0.21</td></tr>
<tr><td>3</td></tr>
<tr><td>4</td></tr>
<tr><td rowspan="3">Tail lattice</td><td rowspan="3">110.46</td><td>2</td></tr>
<tr><td>3</td></tr>
<tr><td>4</td></tr>
<tr><td rowspan="3">Head lattice</td><td rowspan="3">69.54</td><td rowspan="6">3</td><td>1.5</td><td rowspan="6">0.21</td></tr>
<tr><td>3.0</td></tr>
<tr><td>4.5</td></tr>
<tr><td rowspan="3">Tail lattice</td><td rowspan="3">110.46</td><td>1.5</td></tr>
<tr><td>3.0</td></tr>
<tr><td>4.5</td></tr>
<tr><td rowspan="3">Head lattice</td><td rowspan="3">69.54</td><td rowspan="3">3</td><td rowspan="6">1.5</td><td>0.21</td></tr>
<tr><td>0.50</td></tr>
<tr><td>0.70</td></tr>
<tr><td rowspan="3">Tail lattice</td><td rowspan="3">110.46</td><td rowspan="3">4</td><td>0.21</td></tr>
<tr><td>0.50</td></tr>
<tr><td>0.70</td></tr>
<tr><td rowspan="3">Head lattice</td><td rowspan="3">69.54</td><td rowspan="6">3</td><td rowspan="6">1.5</td><td rowspan="6">0.21</td><td>2.065</td></tr>
<tr><td>10.00</td></tr>
<tr><td>30.30</td></tr>
<tr><td rowspan="3">Tail lattice</td><td rowspan="3">110.46</td><td>2.065</td></tr>
<tr><td>10.00</td></tr>
<tr><td>30.30</td></tr>
<tr><td rowspan="3">Head lattice</td><td rowspan="3">69.54</td><td rowspan="6"></td><td rowspan="6">1.5</td><td rowspan="6">0.21</td><td rowspan="6">30.30</td><td>0.80</td></tr>
<tr><td>1.0</td></tr>
<tr><td>1.33</td></tr>
<tr><td rowspan="3">Tail lattice</td><td rowspan="3">110.46</td><td>0.80</td></tr>
<tr><td>1.0</td></tr>
<tr><td>1.33</td></tr>
</table>

1) The materials corresponding to E = 2.065 MPa, 10 MPa, and 30.3 MPa are PDMS[23], FLX9085-DM[34], and TPU, with Poisson's ratios of 0.49, 0.3, and 0.45, respectively.
2) The unit cell size is proportionally scaled by multiplying λ with a width of 12.06 mm and a height of 13.50 mm.

Table S2. Comparison of the proposed robot with representative pipeline-crawling soft robots in literatures

	Design characteristics			Crawling in pipes with air 1)		Crawling in pipes with flowing fluids				Ref.
	Body configuration	Locomotion mode	Number of actuators	Speed [mm s^{-1}]	Load-bearing capacity 2)	Ability	Reynolds number	Drag force [N]	Speed 3) [mm s^{-1}]	
Pneumatic actuation	Hollow shell lattice	Earthworm-like	1	7.38	15.52	Yes	0~5×10^4	7.23×10^{-4}~16.46	7.03~1.2	This work
	Blocks	Earthworm-like	3	6	14.09	No	/	/	/	[29]
				NM 4)	3.29					[31]
				4.23	11.71					[33]
			3	NM	10.85					[34]
	3D lattice		1	14	NM	NM				[36]
Magnetic actuation	Microfiber	Rotation	1	NM	NM	Yes	~22.2	7.83×10^{-9}	0.32	[3]
	Sheet-shaped	Multi-mode		NM	NM		NM	~6×10^{-5}	~2.5	[23]
	Stent-shaped	Rotation		NM	NM		~80.2	3×10^{-4}	0.18	[38]

1) The crawling speeds and load-bearing capability in the pipes with air are measured when the robot crawls in vertical pipes.

2) The load-bearing capacity is measured by the ratio of the carried load to the body weight of the robot.

3) The crawling speeds in the pipes with flowing fluids are measured when the robot crawls against the flow.

4) 'NM' denotes Not Mentioned.

Movie S1 Exploded view of shell-lattice soft robot.

Movie S2 Crawling in pipes with flowing water.

Movie S3 Crawling in various types of pipes.

Movie S4 Crawling with payload.

Movie S5 Untethered crawling.

Movie S6 Potential application 1-Pipeline inspection and obstacle cleaning in concealed pipeline.

Movie S7 Potential application 2-Crawling in flexible ocean pipeline.

References

[1] F. S. Lien, E. Yee, Y. Cheng, *J. Wind. Eng. Ind. Aerod.* **2004**, 92, 117.

[2] V. Vullo, *"Thin-Walled Circular Cylinders Under Internal and/or External Pressure and Stressed in the Linear Elastic Range" in Circular cylinders and pressure vessels: Stress Analysis and Design*, Springer, Switzerland **2014**.
